# Transcriptome Analysis Provides Insights into the Mechanisms Underlying Wheat Plant Resistance to Stripe Rust at the Adult Plant Stage

**DOI:** 10.1371/journal.pone.0150717

**Published:** 2016-03-18

**Authors:** Yingbin Hao, Ting Wang, Kang Wang, Xiaojie Wang, Yanping Fu, Lili Huang, Zhensheng Kang

**Affiliations:** 1 State Key Laboratory of Crop Stress Biology for Arid Areas and College of Plant Protection, Northwest A&F University, Yangling, PR China; 2 State Key Laboratory of Crop Stress Biology for Arid Areas and College of Life Science, Northwest A&F University, Yangling, PR China; Cankiri Karatekin University, TURKEY

## Abstract

Stripe rust (or yellow rust), which is caused by *Puccinia striiformis* f. sp. *tritici* (*Pst*), is one of the most devastating wheat diseases worldwide. The wheat cultivar Xingzi 9104 (XZ) is an elite wheat germplasm that possesses adult plant resistance (APR), which is non–race-specific and durable. Thus, to better understand the mechanism underlying APR, we performed transcriptome sequencing of wheat seedlings and adult plants without *Pst* infection, and a total of 157,689 unigenes were obtained as a reference. In total, 2,666, 783 and 2,587 differentially expressed genes (DEGs) were found to be up- or down-regulated after *Pst* infection at 24, 48 and 120 hours post-inoculation (hpi), respectively, based on a comparison of *Pst*- and mock-infected plants. Among these unigenes, the temporal pattern of the up-regulated unigenes exhibited transient expression patterns during *Pst* infection, as determined through a Gene Ontology (GO) enrichment analysis. In addition, a Kyoto Encyclopedia of Genes and Genomes (KEGG) pathway analysis showed that many biological processes, including phenylpropanoid biosynthesis, reactive oxygen species, photosynthesis and thiamine metabolism, which mainly control the mechanisms of lignification, reactive oxygen species and sugar, respectively, are involved in APR. In particular, the continuous accumulation of reactive oxygen species may potentially contribute to the ability of the adult plant to inhibit fungal growth and development. To validate the bioinformatics results, 6 candidate genes were selected for further functional identification using the virus-induced gene silencing (VIGS) system, and 4 candidate genes likely contribute to plant resistance against *Pst* infection. Our study provides new information concerning the transcriptional changes that occur during the *Pst*-wheat interaction at the adult stage and will help further our understanding of the detailed mechanisms underlying APR to *Pst*.

## Introduction

Stripe rust (or yellow rust), which is caused by *Puccinia striiformis* f. sp. *tritici* (*Pst*), is a common and damaging disease of wheat *(Triticum aestivum* L.) that causes significant yield and grain quality losses [1–4]. The plant innate immune response is highly diverse in its capacity to recognize and respond to biotrophs, and most plants are resistant to most plant pathogens [5]. Accordingly, the breeding of resistant cultivars is the most effective, economic and environmentally favorable method for controlling diseases [6]. Stripe rust resistance is broadly categorized as either all-stage resistance (seedling resistance) or adult plant resistance (APR) [1]. Because that *Pst* rapidly evolves new races, cultivars with seedling resistance to *Pst* usually become susceptible within a few years after being popularized [7, 8]. However, wheat cultivars with APR usually remain resistant even after being popularized in large areas for many years [9, 10]. Wheat cultivar Xingzi 9104 (XZ) is susceptible to *Pst* at seedling stage, which possesses non-race specific to stripe rust at adult plant stage [4, 11–13]. However, its underlying protective mechanism is still unclear. Over the past several years, the rapid and inexpensive next-generation sequencing (NGS) method has resulted in high-throughput gene expression profiling, genome annotation and the discovery of non-coding RNA [14, 15]. An increasing body of evidence suggests that transcriptome sequencing using NGS technology provides high-resolution data and is a powerful tool for studying global transcriptional networks. Using this technology, we can successfully obtain a large amount of sequence data that may represent the gene expression profile of wheat under a particular condition. To the best of our knowledge, the transcription profiling of the wheat cultivar XZ infected with stripe rust has not yet been reported. Considering the agricultural value of XZ, a comprehensive description of the genes expressed in XZ during infection is necessary for the discovery of candidate defense-related genes. In this study, we obtained a large number of distinct sequences (designated as unigenes) from an equal mix of total RNA from XZ at 0 hours post-inoculation (hpi) without *Pst* at the adult plant stage (Ak-M-0) and total RNA from XZ at 0 hpi without *Pst* at the seedling stage (Sk-M-0) using the Illumina NGS technology. In this manuscript, we present the current understanding of the assembled and annotated transcriptome sequences. Additionally, we provide information obtained from a digital gene expression (DGE) system that was used to compare the gene expression profiles of adult XZ plants at three infection stages, namely 24, 48 and 120 hpi with *Pst*. This comparison allowed us to elucidate the molecular mechanism and identify the responsive genes underlying the complex XZ resistance to *Pst*. These findings should facilitate the development of effective strategies for the breeding of resistant “wheat” varieties to obtain a better control of stripe rust.

## Materials and Methods

### Plant materials and *Pst* inoculation

*Triticum aestivum* cv. XZ and *Pst* pathotype CYR32 were used in the study. For the preparation of plant samples, germinated seeds were maintained at 4°C for 40 days for vernalization before being planted in large 20-cm-diameter pots, which contained mellow soil. These pots, which contained five seeds, were placed in a greenhouse at 20°C for cultivation, where they received 16 hours of light a day. Topdressing can be carried out with water during booting stage of wheat. For the inoculation, the second leaf of wheat seedling plants at the two-leaf stage and the flag leaf of wheat adult plants at the boot stage were simultaneously inoculated with fresh *Pst* race CYR32 with a paintbrush until whole surface became wet without run-off as described [12]. Control plants (mock-inoculated plants) were treated with sterile water. Inoculated and control leaves were collected at 0, 24, 48 and 120 hpi for RNA isolation. These time points were selected based on microscopic studies [12]. All of the samples were rapidly frozen in liquid nitrogen and stored at -80°C. The remaining plants were rated in terms of symptoms at 15 days post-inoculation (dpi). Three independent biological replicates were performed for each time point.

### RNA isolation and cDNA synthesis

The total RNA was isolated using lysis buffer from the RNeasy Plant Mini Kit according to the manufacturer’s instructions (QIAGEN, Hilden, Germany). DNA was removed using the TRIzol Reagent TURBO (Qiagen RNase-Free DNase set) (Ambion) according to the manufacturer’s instructions. The RNA integrity was confirmed by 1.0% agarose gel electrophoresis, and the total RNA quantity was determined with a NanoDrop 1000 spectrophotometer (Thermo Fisher Scientific, Waltham, MA, USA). First-strand cDNA was synthesized with 2.5 μg of total RNA using the Reverse Transcription System (Promega, Madison, WI, USA) following the manufacturer’s directions.

### Illumina library construction and sequencing

The RNA of two samples (Ak-M-0 and Sk-M-0) was subjected to RNA-Seq analysis at the Beijing Genomics Institute (BGI; Shenzhen, China). The transcriptome library was prepared and sequenced using Illumina HiSeq^TM^2000 using paired-end technology. A fragmentation buffer was used to cut the mRNA into short fragments (approximately 200 bp). These short fragments were used as templates with random hexamer-primers to synthesize the first-strand cDNA. The second-strand cDNA was synthesized using buffer, dNTPs, RNaseH and DNA polymerase I. Short double-stranded cDNA fragments were purified using a QIAquick PCR purification kit (Qiagen) and resolved with EB buffer for end repair prior to the addition of poly(A). The short fragments were then connected with sequencing adaptors. We then selected suitable fragments as templates via agarose gel electrophoresis and enriched them by PCR amplification. A Solexa HiSeq^TM^2000 sequencer was employed to sequence the constructed libraries [16].

### DGE library construction and sequencing

The RNA of six samples (Ak-M-24, Ak-M-48, Ak-M-120; Ak-I-24, Ak-I-48 and Ak-I-120) was prepared for DGE library construction and sequencing at the Beijing Genomics Institute (BGI; Shenzhen, China). In brief, the mRNA was purified using oligo (dT) magnetic beads. The first- and second-strand cDNA were synthesized after the mRNA was bound to the beads. While on the beads, the double-stranded cDNA was then digested with the anchoring restriction enzyme *NlaIII* to remove all fragments other than the 3’-most CATG fragment attached to the oligo bead, and the GEX adapter 1 was added to the new 5’end. The junction of GEX adapter 1 and the CATG site was recognized by *MmeI*. This enzyme cuts 17 bp downstream of the CATG site, thus producing 17-bp cDNA sequence tags with GEX adapter1. After removing the 3’ fragments through magnetic bead precipitation, GEX adapter 2 was ligated to the 3’ end of the cDNA tag. Tags flanked by both adapters were enriched by PCR using the GEX PCR primers 1 and 2 (Illumina) according to the manufacturer’s instructions. The PCR products were purified with a 12% PAGE gel. The purified cDNA tags were sequenced on an Illumina cluster station and genome analyzer (Illumina) following the manufacturer’s instructions [17, 18].

### Raw read cleaning, assembly and sequence annotation

Prior to assembly, raw reads were obtained from the original sequence data, and the reads were filtered using a Perl script dynamic-Trim.pl [19] to remove the adaptor sequences, empty reads, short reads (<25bp), reads with an N ratio greater than 10%, and low-quality sequences, all of which negatively affect the bioinformatics analysis. We then generated clean reads in the FASTQ format. All of the clean reads were mapped to our transcriptome reference database, allowing no more than a 2-bp mismatch using the software SOAP aligner/soap2. The results revealed the distribution and location of the clean reads in the reference genome. *De novo* assembly of the high-quality reads was performed using SOAP *de novo* and yielded unigenes. The unigenes of two samples were spliced and processed by clustering software to obtain the longest possible non-redundant unigene. Finally, the generated unigenes were analyzed by a BlastX alignment search (E-value<10^−5^) against the protein databases NR, Swiss-Prot, KEGG (Kyoto Encyclopedia of Genes and Genomes), and GO (Gene Ontology), and the best aligning result was used to determine the sequence unigenes direction [16, 20].

### Statistical analysis of gene expression levels

Gene expression was calculated from the number of reads mapped to the reference sequence [21]. The expression level was calculated using the RPKM method (reads per kb per million reads) with the following formula:

$$RPKM=\frac{10^{6}C}{NL/10^{3}}$$

where RPKM (A) is the expression of gene A, C is the number of reads uniquely aligned to gene A, N is the total number of reads uniquely aligned to all of the genes, and L is the number of bases on gene A. The RPKM method eliminates the influence of different gene lengths and sequencing discrepancies during the calculation of gene expression such that the calculated gene expression levels can be directly compared among samples. If there is more than one transcript for a gene, the longest is used to calculate its expression level and coverage [22].

### Evaluation of DGE libraries

We compared the differential expression of genes in each DGE library (Ak-I-24 vs. Ak-M-24, Ak-I-48 vs. Ak-M-48 and Ak-I-120 vs. Ak-M-120) using the method described by Audic and Claverie [23]. The correlation between the detected count numbers between parallel libraries was assessed statistically by calculating Pearson’s correlation (*P*) coefficient. In addition to the *P* value, the false discovery rate (FDR) was used to determine the threshold *P* value in multiple tests. Genes were classified as significantly differentially expressed if they had a *P*-value less than 0.005, an FDR less than 0.001 and an absolute value of log_2_Ratio of at least 1 in sequence counts across the libraries utilized in our study.

### GO and KEGG pathway enrichment analysis

GO enrichment analysis provides all of the GO terms that are significantly enriched in differentially expressed genes (DEGs) compared with the genome background and filters the reads that correspond to biological functions. This method first maps all DEGs to GO terms in the database by calculating gene numbers for every term and then uses a hypergeometric test to identify the significantly enriched GO terms in the DEGs compared with the genome background. The KEGG database was used to identify the significantly enriched metabolic pathways or signal transduction pathways in the DEGs compared with whole genome backgrounds. N is the number of all of the genes with a KEGG annotation, n is the number of DEGs in N, M is the number of all of the genes annotated to specific pathways, and m is the number of DEGs in M.

### Quantitative real-time PCR analysis

Quantitative real-time PCR (qRT-PCR) was performed to verify the expression of 30 differentially expressed genes. To standardize the data, the wheat translation elongation factor 1α subunit gene (TaEF-1α, GenBank Accession Number M90077.1) was used as an internal reference for the qRT-PCR analysis. The qRT-PCR analysis was performed in triplicate using the SYBR Green fluorescence dye in a 7500 Real-Time PCR System (Applied Biosystems, Foster City, CA, USA). Each qRT-PCR reaction (25 μL) included 2.5 μL of 10×Taq Buffer, 3.0 μL of MgCl_2_ (25 mM/L), 0.5 μL of dNTPs (10 mM/L), 0.4 μL of 50×SYBR Green (QIAGEN, Hilden, Germany), 2.0 μL of cDNA, 0.5 μL of forward/reverse primers (10 μmol/L) and 0.3 μL of Taq DNA polymerase (TaKaRa Bio Inc., Japan). The qRT-PCR data were analyzed using the comparative 2^-ΔΔCt^ method [24]. Mean and standard deviation were calculated with data from 3 independent biological replicates.

### Histological observations and host response

Capped *in vitro* transcripts were prepared from linearized recombinant plasmids containing the tripartite BSMV genome [25] with the mMessage mMachine T7 *in vitro* transcription kit (Ambion, Austin, TX, USA) according to the manufacturer's guidelines. Three independent sets of plants were used, and germinated seeds were maintained at 4°C for 40 days for vernalization before being planted in large pots. The inoculation system contained 0.5 μL of RNAs for each *in vitro* transcription reaction for α, β, and γ (γ-PDS, γ-gene) (phytoene desaturase) and was gently rubbed on the surface with 25 μL of FES buffer. The mixtures were inoculated on the surface of the second top leaves of boot stage plants with a gloved finger [26, 27], and the plants were then incubated at 23±2°C. The virus phenotypes were observed and photographed 13 days after virus inoculation. The flag leaves were further inoculated with *Pst* pathotype CYR32, and samples were excised at 0, 24, 48 and 120 hpi for histological observation and qRT-PCR. The *Pst* infection phenotypes were recorded and photographed at 15 dpi. The samples for histological observation were fixed and decolorized as previously described [28]. The necrotic area in wheat leaves was observed via the auto fluorescence of the attacked mesophyll cells by Olympus BX-51 microscope (Olympus Corp., Japan) and calculated by the cellSens Entry software (Olympus Corp., Japan). To stain the infection structures of *Pst* in wheat leaves, wheat germ agglutinin (WGA) conjugated to Alexa-488 (Invitrogen, Carlsbad, CA, USA) was used as previously described [29]. For microscopic observations, stained segments were kept in 50% glycerol, and the hyphal length was examined under blue light excitation (excitation wavelength 450–480 nm, emission wavelength 515 nm) by Olympus BX-51 microscope (Olympus Corp., Japan) as previously described [29]. To detect host response, hydrogen peroxide accumulation was detected using 3, 3'-diaminobenzidine (DAB; Amresco, Solon, OH, USA) as previously described [29]. At least 30–50 infection sites were examined on each of three randomly selected leaf segments for every treatment. A high performance liquid chromatographic method was used to determine the endogenous hormones salicylic acid (SA) content in flag leaves as previously described [30]. The lignin content of the flag leaves was examined according to the method of Morrison with some modifications as previously described [31]. The chloroplast content of the flag leaves was determined using the direct sopping extraction method with mixing solution of alcohol and acetone (1:1 in volume) as previously described [32].

## Results

### High-throughput RNA sequencing

The transcriptome sequencing of seedling and adult plants samples at 0 hpi resulted in a total of 66,666,668 and 63,954,974 reads, respectively. The ratio of the Q20 sequencing value was greater than 91%, which indicated that the sequencing sufficiently captured most of the expressed genes (Table 1). Transcriptome *de novo* assembly was conducted with the Short Oligonucleotide Analysis Package (SOAP) program and resulted in 1,157,689 specific unigenes (S1 Fig). For the functional annotation and classification of the obtained unigenes, we searched the annotated sequences for genes that were involved in the cluster of orthologous group (COG) assignments (S2 Fig). The sequences could be categorized into 35 level-two functional groups, which comprised three domains: ‘biological process’, ‘cellular component’ and ‘molecular function’ (S3 Fig). A G-test [False Discovery Rate (FDR) < 0.001] of the RPKM-derived read counts with multiple genetic differences greater than two-fold was performed to detect the DEGs between the adult plant (Ak-M-0) and seedling (Sk-M-0) stages and to identify the genes responsible for the development of wheat (S4 Fig). A total of 27,265 DEGs were obtained from this analysis; of these, 13,977 and 13,288 unigenes were up- and down-regulated at the adult plant stage respectively, which suggests that these genes may be involved in the growth and development of the host plant.

**Table 1:** Output statistics from sequencing.

Samples	Total Reads	Total Nucleotides	Q20 percentage	N percentage	GC percentage
AK-M-0	66,666,668	6,000,000,120	91.74	0.00	53.96
SK-M-0	63,954,974	5,755,947,660	91.57	0.00	54.32

Total Nucleotides = Total Reads1 × Read1 size + Total Reads2 × Read2 size. The two libraries included non-inoculated adult plants at 0 hours post-inoculation (hpi) (Ak-M-0) and non-inoculated seedling plants at 0 hpi (Sk-M-0).

### DGE library sequencing and annotation

**Fig 1:**
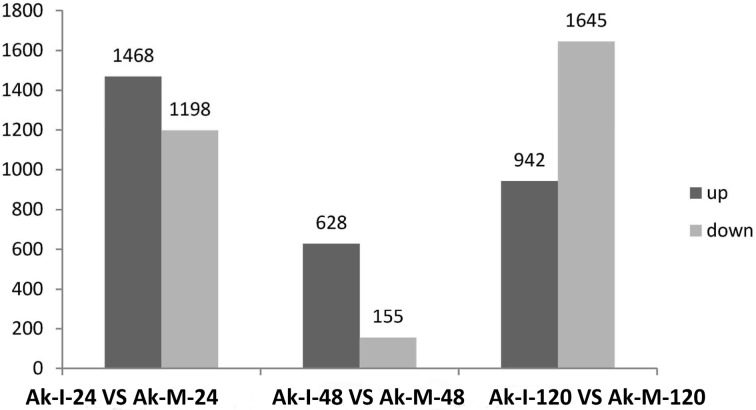
Statistical chart of DEGs during *Pst* infection at the adult plant stage. Differentially expressed genes were identified by filtering the two-fold up-regulated and down-regulated genes with FDR≤10^−4^. The bars represent the number of up-regulated (black) and down-regulated (gray) unigenes. The six DGE libraries included non-inoculated adult plants at 24 hours post-inoculation (hpi) (Ak-M-24), 48 hpi (Ak-M-48) and 120 hpi (Ak-M-120), and inoculated adult plants at 24 hpi (Ak-I-24), 48 hpi (Ak-I-48) and 120 hpi (Ak-I-120).

**Fig 2:**
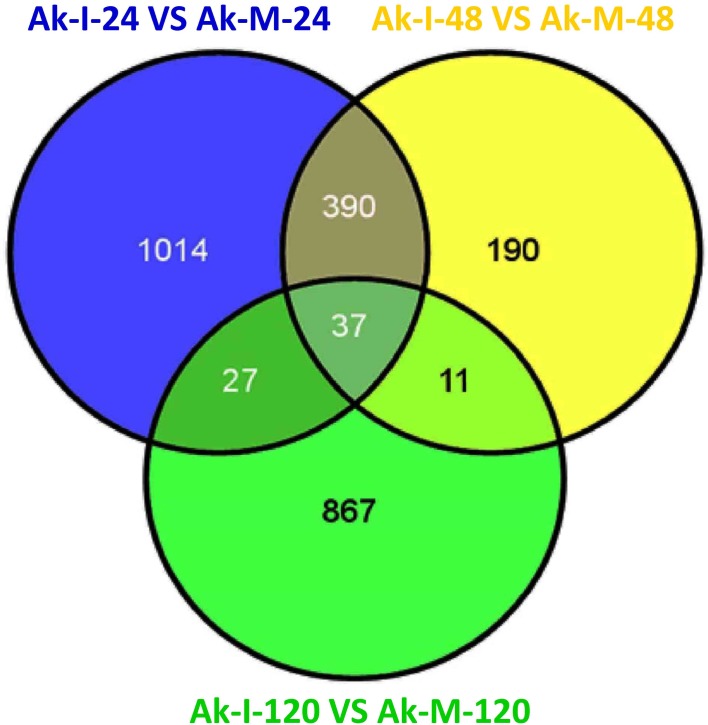
Statistical chart of DEGs up-regulated during *Pst* infection at the adult plant stage. Differentially expressed unigenes were identified by filtering the two-fold up-regulated unigenes with FDR≤10^−4^. The up-regulated unigenes were obtained from the Ak-I-24 vs. Ak-M-24, Ak-I-48 vs. Ak-M-48 and Ak-I-120 vs. Ak-M-120 comparisons. The six DGE libraries included non-inoculated adult plants at 24 hours post-inoculation (hpi) (Ak-M-24), 48 hpi (Ak-M-48) and 120 hpi (Ak-M-120), and inoculated adult plants at 24 hpi (Ak-I-24), 48 hpi (Ak-I-48) and 120 hpi (Ak-I-120).

**Fig 3:**
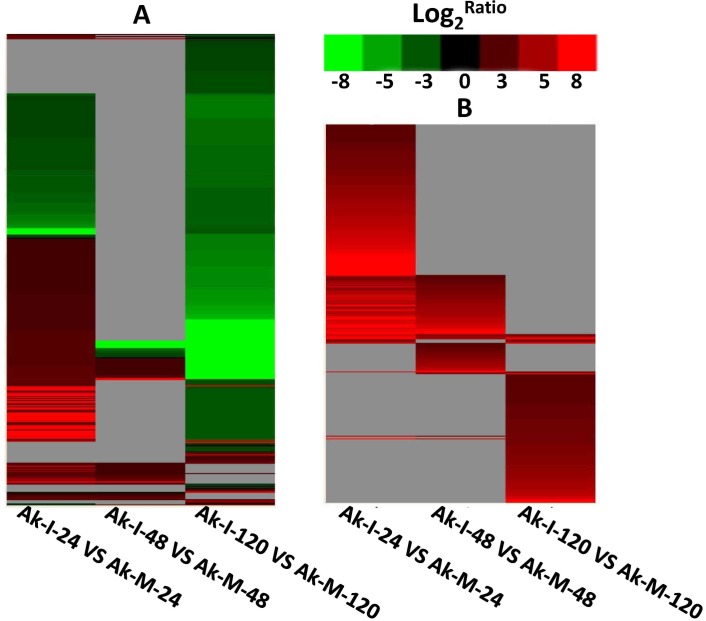
Cluster and heat map of DEGs of XZ at the adult plant stage. The heat map shows the gene expression obtained by the clustering affinity search technique. Each line refers to the data for one gene. The color bar represents the log2 of fold change values and ranges from green (−8) to red (8). The six DGE libraries included non-inoculated adult plants at 24 hours post-inoculation (hpi) (Ak-M-24), 48 hpi (Ak-M-48) and 120 hpi (Ak-M-120), and inoculated adult plants at 24 hpi (Ak-I-24), 48 hpi (Ak-I-48) and 120 hpi (Ak-I-120). A: The DEGs that demonstrated at least a two-fold difference in each of the three comparisons at the adult plant stage. B: The up-regulated unigenes that demonstrated at least a four-fold change in each of the three comparisons at the adult plant stage.

Based on the transcriptome sequence data, six DGE libraries were constructed to identify the gene expression profiles of the XZ during *Pst* infection at the adult plant stage. The six DGE libraries included non-inoculated adult plants at 24 hpi (Ak-M-24), 48 hpi (Ak-M-48) and 120 hpi (Ak-M-120) and inoculated adult plants at 24 hpi (Ak-I-24), 48 hpi (Ak-I-48) and 120 hpi (Ak-I-120). All of the clean reads were mapped to our transcriptome reference database. Each library generated raw reads that ranged from 290 to 318 Mb, with reference genome alignments greater than 84% (S1 Table). Together, each library generated clean reads that ranged from 5.78 to 6.34 million, and the proportion of the total reads exceeded 98%, which indicates that the transcriptional data were reliable (S5 Fig). Saturation analyses of the six DGE libraries were performed to determine whether the number of detected genes continued to increase as the sequence quantity increased (total tag number). The number of detected genes almost ceased to increase at a sequence quantity of at least 600 million (S6 Fig); the coverage statistics were high (S7 Fig). All of these findings indicate that the sequencing data was accurate and reliable. A G-test (FDR<0.001) of the RPKM-derived read counts with multiple genetic differences greater than four-fold was performed to detect the DEGs within these pairs, i.e., (Ak-I-24 vs. Ak-M-24), (Ak-I-48 vs. Ak-M-48) and (Ak-I-120 vs. Ak-M-120), and to identify genes that are responsive to *Pst* infection at the adult plant stage. Approximately 2,666, 786 and 2,587 DEGs were obtained at 24, 48 and 120 hpi, respectively. Additionally, 1,198, 155 and 1,645 unigenes were down-regulated and 1,468, 628 and 942 unigenes were up-regulated at 24, 48 and 120 hpi, respectively (Fig 1). A total of 427 unigenes were up-regulated between 24 and 48 hpi, whereas 48 unigenes were up-regulated between 48 and 120 hpi. In addition, 64 unigenes were up-regulated between 24 and 120 hpi, and 37 unigenes were up-regulated at all three sampled stages (Fig 2). The regulated unigenes represented on the array were significantly differentially expressed at 24, 48 and 120 hpi. We used the gene cluster set to generate a tree that shows the similarities in the relative gene expressions among the three time points (Fig 3).

### Regulated genes during *Ps*t infection at the adult plant stage

**Fig 4:**
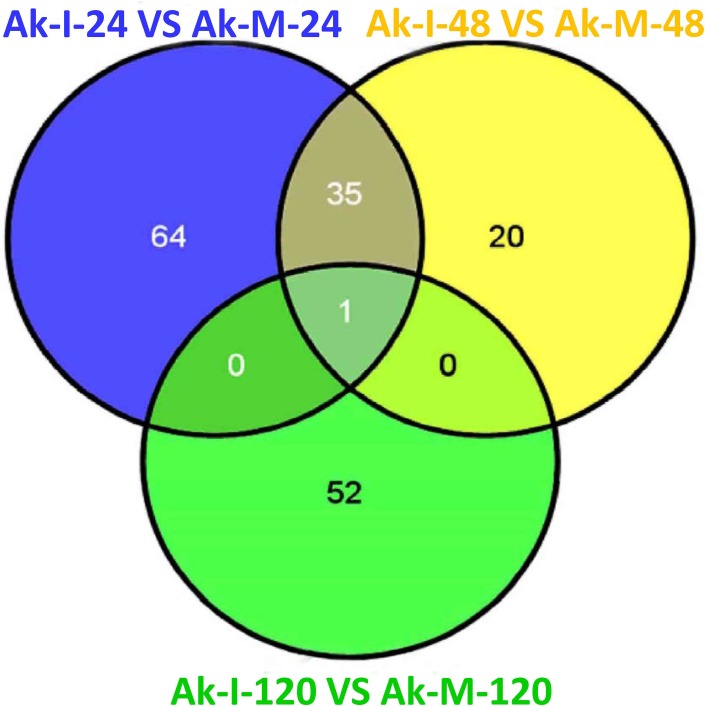
Statistical chart of enrichments in ‘biological processes’ during *Pst* infection at the adult plant stage. The leaves of adult plants were inoculated with *Pst* CYR32. The panels represent the transcriptional changes in ‘biological processes’ obtained from the Ak-I-24 vs. Ak-M-24, Ak-I-48 vs. Ak-M-48 and Ak-I-120 vs. Ak-M-120 comparisons. The six DGE libraries included non-inoculated adult plants at 24 hours post-inoculation (hpi) (Ak-M-24), 48 hpi (Ak-M-48) and 120 hpi (Ak-M-120), and inoculated adult plants at 24 hpi (Ak-I-24), 48 hpi (Ak-I-48) and 120 hpi (Ak-I-120).

To investigate the plant disease-resistance of XZ during *Pst* infection, Fisher’s exact test in the Blast2GO software was used to explore the statistically enriched GO terms of the up-regulated genes during *Pst* infection at the adult plant stage compared with the entire transcriptome background (*P*<0.05). The results of the up-regulated unigenes identified from the Ak-I-24 vs. Ak-M-24 comparison were as follows. In the ‘cellular component’ category, proteins involved with cytoplasmic membrane-bounded vesicles, the chloroplastic endopeptidase Clp complex, the glyoxysome and the cell wall were highly enriched. In the ‘molecular function’ category, proteins involved in phenylalanine ammonia-lyase activity, heme binding and pyruvate and phosphate dikinase activity were highly enriched, and 78 compounds were also enriched. In the ‘biological process’ category, proteins involved in the SA catabolic process, cinnamic acid biosynthetic process and L-phenylalanine catabolic process were highly enriched, and an additional 97 compounds were also enriched (S1 File). The results from the up-regulated unigenes identified from the Ak-I-48 vs. Ak-M-48 comparison and the Ak-I-120 vs. Ak-M-120 comparison are as follows (S1 File). Thirty-six categories were enriched in the Ak-I-24 vs. Ak-M-24 and Ak-I-48 vs. Ak-M-48 comparisons (Fig 4). Only one category was enriched in the Ak-I-48 vs. Ak-M-48 and Ak-I-120 vs. Ak-M-120 comparisons, whereas 36 categories were enriched in the Ak-I-24 vs. Ak-M-24, Ak-I-48 vs. Ak-M-48 and Ak-I-120 vs. Ak-M-120 comparisons (Fig 4).

### KEGG pathways are differentially expressed in response to *Pst*

To explore the biochemical pathways in which the up-regulated DEGs identified in the Ak-I-24 vs. Ak-M-24, Ak-I-48 vs. Ak-M-48 and Ak-I-120 vs. Ak-M-120 comparisons are involved in XZ wheat, a pathway analysis utilizing the KEGG pathway database was conducted with an E-value cutoff of < 0.05. Interestingly, the KEGG pathway analysis showed that 45, 42 and 34 significantly enriched pathways were identified at the three infection stages, namely 24 hpi, 48 hpi and 120 hpi, respectively (S2 File). These up-regulated DEGs were found to be involved in many biological processes related to systemic symptom development, including thiamine metabolism, purine metabolism, phenylpropanoid biosynthesis, novobiocin biosynthesis and photosynthesis. An additional 15 significantly enriched pathways were identified at the three adult plant stages. Fourteen significantly enriched pathways were only identified from the Ak-I-24 vs. Ak-M-24 comparison, whereas 5 significantly enriched pathways were only identified in the Ak-I-48 vs. Ak-M-48 comparison, and 7 significantly enriched pathways were only identified from the Ak-I-120 vs. Ak-M-120 comparison.

### Comparison of digital gene expression values with qRT-PCR analysis results

To evaluate the reliability of our RNA-Seq and DGE analysis, 30 unigenes, which presented a wide range of expression levels and patterns under *Pst* infection, were selected for qRT-PCR analysis. These unigenes were selected based on their homology to genes that are known to play a role in pathogenesis/defense, reactive oxygen species (ROS) burst, secondary metabolism or that encode hypothetical or unknown proteins. The corresponding primers were designed and are listed in S3 File. As shown in Table 2, the results agreed well with the DGE patterns. In addition, the unigenes involved in pathogenesis-related proteins (wheat11332_refgene, wheat10510_refgene and wheat59172_refgene) were strongly up-regulated as early as 24 hpi and afterward presented relatively up-regulated expression. The transcripts of wheat31335_refgene and wheat12328_refgene were increased by 3.53 to 21.09 -fold, respectively. The unigenes of wheat57563_refgene, wheat12902_refgene, wheat75137_refgene, wheat75952_refgene and wheat10297_refgene, which are related to ROS burst, presented up-regulated expression at 24, 48 and 120 hpi. However, 6 unigenes with putative functions associated with thiol methyltransferase 2, ATP synthase subunit alpha, chloroplastic, protease Do-like 2, ribonuclease1 and a hypothetical protein were down-regulated as early as 24 and 48 hpi. The remaining 15 unigenes showed significantly higher expression patterns, which suggests that the selected unigenes may play an active role during the interaction between wheat and *Pst*.

**Table 2:** Verification of DGE-Seq results by qRT-PCR.

Gene Name	Annotation (BLASTX)	Relative gene expression by qRT-PCR ^(2− ΔΔCt)^			Gene expression of Illumina (Log2^(T_RPKM/C_RPKM)^)		
		24hpi	48hpi	120hpi	24hpi	48hpi	120hpi
wheat8730_refgene	beta-fructofuranosidase	2.02±0.31	3.21±0.52	3.09±0.43	1.10	1.50	1.55
wheat84218_refgene	thaumatin-like protein	20.99±5.55	6.88±1.01	6.02±0.77	5.59	2.16	1.37
wheat7284_refgene	beta-1,3-glucanase	30.22±3.53	7.09±1.11	8.77±1.92	4.32	1.72	1.66
wheat72566_refgene	predicted protein	3.83±0.89	2.01±0.54	5.76±0.32	1.12		2.15
wheat33491_refgene	predicted protein	33.23±3.09	7.42±2.09	10.09±3.78	5.18	1.62	2.82
wheat31335_refgene	Cell wall-associated hydrolase	5.88±1.73	3.53±2.01	3.86±0.22	1.15		1.77
wheat12328_refgene	class I chitinase	21.09±3.22	4.54±0.35	9.78±2.98	4.12	1.03	3.12
wheat121909_refgene	Glutathione S-transferase 2	9.88±1.78	1.09±0.88	5.54±1.63	2.59		2.09
wheat10297_refgene	root peroxidase	3.09±0.77	9.09±3.02	2.00±1.09	1.24	2.40	
wheat14242_refgene	hypothetical protein	-7.33+1.11	-2.99±0.98	4.87±0.86	-2.57	-1.10	1.17
wheat8458_refgene	predicted protein	5.32±1.99	7.09±2.01	-1.12±0.79	1.60	1.16	-1.46
wheat13698_refgene	unnamed protein product	4.09±6.99	2.44±5.00	2.56±2.01	2.06	1.82	1.47
wheat8254_refgene	Ribonuclease 1	-7.09±1.22	-5.66±0.99	-9.08±1.33	-2.34	-1.64	-3.52
wheat7959_refgene	3-beta-hydroxysteroid-dehydrogenase	11.03±2.09	6.08±0.99	15.88±2.75	3.65	2.46	3.49
wheat75952_refgene	lipoxygenase 1.1	3.21±0.94	4.00±0.86	-12.09±3.02	1.51	1.41	-3.33
wheat75846_refgene	putative thiol methyltransferase 2	-22.09±3.33	-5.86±1.03	5.99±1.21	-4.12	-1.77	1.19
wheat7310_refgene	MAP kinase	4.32±0.67	3.98±0.99	-5.00±1.81	1.52	1.36	-1.48
wheat70819_refgene	ATP synthase subunit alpha, chloroplastic	-2.74±0.97	-3.03±1.02	-4.23±0.56	-1.29	-1.08	-1.72
wheat66652_refgene	Protein WIR1A	8.23±1.83	9.09±0.86	7.99±1.24	2.79	2.43	2.24
wheat59172_refgene	pathogenesis-related 5	24.35±3.80	8.79±2.43	5.79±0.75	4.73	2.38	1.60
wheat57862_refgene	hypothetical protein F775_31113	6.08±1.00	3.08±0.69	7.99±1.80	2.33	1.88	2.36
wheat57563_refgene	NAD(P)H-dependent oxidoreductase 1	5.09±0.84	6.32±0.79	5.09±0.69	1.24	1.43	2.03
wheat11332_refgene	pathogenesis-related protein 1	13.98±2.11	4.09±0.34	5.89±0.44	3.97	1.90	1.76
wheat10510_refgene	pathogenisis-related protein 1.1	7,67±0.68	3.90±0.74	4.10±1.02	2.94	1.49	1.46
wheat37392_refgene	Annexin D1	3.09±0.99	1.09±0.88	2.10±0.99	1.57		
wheat12902_refgene	class III peroxidase	8.09±0.56	1.07±1.10	1.90±0.79	2.93		
wheat75137_refgene	Peroxidase 12	155.88±9.38	1.03±0.33	0.67±0.44	13.94		
wheat31306_refgene	predicted protein	3.99±0.52	-0.33±0.22	0.24±0.13	1.89		
wheat36302_refgene	hypothetical protein F775_32419	-0.77±0.25	-0.18±0.03	4.09±0.51			1.13
wheat12266_refgene	Protease Do-like 2	-0.42±0.11	-0.33±0.47	3.08±0.39			1.00

### Functional analysis of six candidate genes in the resistance response to *Pst*

**Fig 5:**
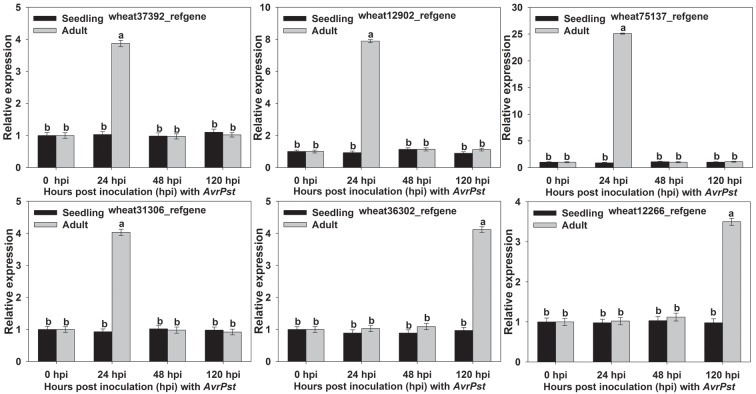
Quantitative real-time polymerase chain reaction analysis of the relative transcript levels of the six candidate unigenes induced by *Pst* infection at seedling and adult stages. The relative expression levels of the unigenes were calculated by the comparative threshold method (2^–ΔΔCT^) and were relative to that at the 0 hour time point. The results are presented as the means ± standard errors of three biological replications. The different letters represent significant differences [*P*≤0.05 according to analysis of variance (ANOVA)].

**Fig 6:**
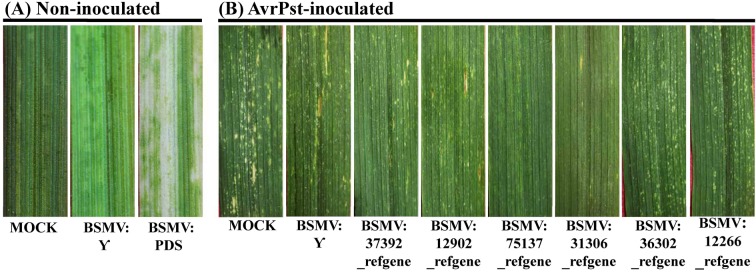
Phenotypes of wheat leaves in candidate gene-knockdown plants inoculated with *Pst*. To perform a functional analysis of the candidate genes, virally induced gene silencing (VIGS) was applied to adult plants. (A) No change was found in mock-inoculated wheat leaves pre-inoculated with FES buffer; mild chlorotic mosaic symptoms were observed on the flag leaves of wheat at 13 days post-inoculation (dpi) with BSMV:γ; photobleaching was evident on the first top leaves of plants 15 days after infection with BSMV:PDS. (B) Mock-inoculated wheat flag leaves treated with FES buffer and then challenged with *Pst* CYR32; the flag leaves of knockdown plant (BSMV:γ, BSMV:wheat37392_refgene, BSMV:wheat12902_refgene, BSMV:wheat75137_refgene, BSMV:wheat31306_refgene, BSMV:wheat36302_refgene and BSMV:wheat12266_refgene) challenged with *Pst* CYR32. Typical leaves were observed and photographed at 15 dpi.

**Fig 7:**
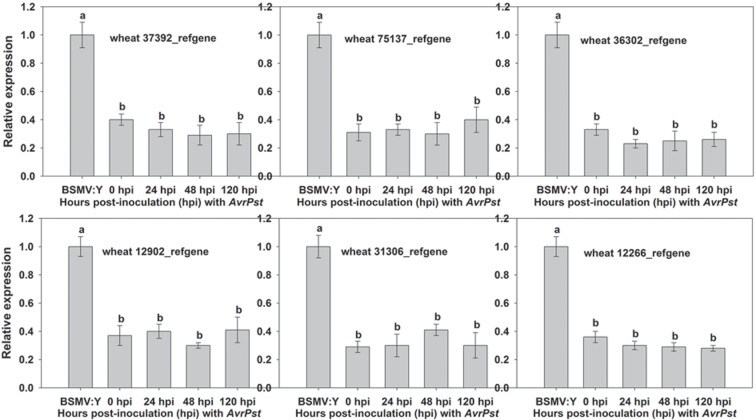
Relative transcript levels of candidate genes in candidate gene-knockdown wheat leaves. RNA samples were isolated from the flag leaves of wheat infected with BSMV:γ, BSMV:wheat37392_refgene, BSMV:wheat12902_refgene, BSMV:wheat75137_refgene, BSMV:wheat31306_refgene, BSMV:wheat36302_refgene and BSMV:wheat12266_refgene at 0, 24, 48 and 120 hours post-inoculation (hpi) with *Pst* CYR32. The error bars represent the variations among three independent replicates. The different letters represent significant differences [*P*≤0.05 according to analysis of variance (ANOVA)]. The relative gene expression levels were quantified using the comparative threshold (2^-ΔΔCT^) method and compared with that of BSMV:γ.

**Fig 8:**
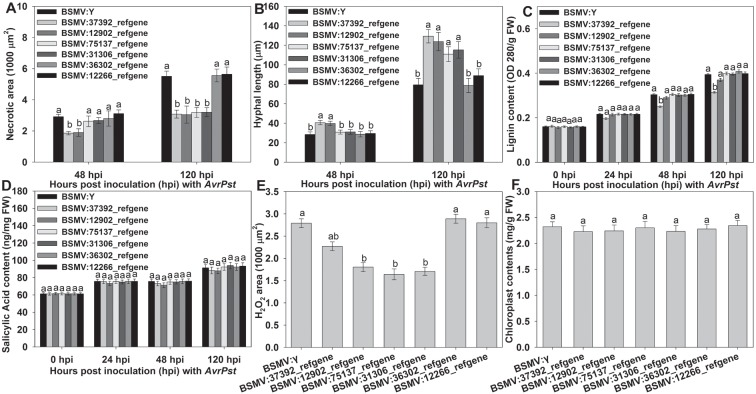
Functional analysis of six candidate genes during the interaction between XZ and stripe rust using the BSMV-mediated virus-induced gene silencing system. The second top leaves were pre-inoculated with BSMV:γ or recombinant BSMV followed by inoculation with *Puccinia striiformis* f. sp. *tritici* race CYR32. (A) Necrotic area, the average area was calculated by DP-BSW software. wheat37392_refgene, wheat12902_refgene, wheat75137_refgene or wheat31306_refgene -silenced plants showed a significant decrease at 120 hours post-inoculation (hpi). (B) Hyphal length, the length of IH was measured from the substomatal vesicle to the apex of the longest infection hyphae, Wheat-37392_refgene, wheat12902_refgene, wheat75137_refgene and wheat31306_refgene -silenced plants showed a significant increase at 120 hpi. (C) Wheat37392_refgene-silenced plants showed a significant decrease in the content of lignin at 48 and 120 hpi. (D) Wheat- 37392_refgene or 12902_refgene -silenced plants showed a decrease in the content of SA at 48 and 120 hpi, but no significant decrease between control and silenced plants. (E) Significant decrease in ROS accumulation in wheat-12902_refgene, wheat-75137_refgene and wheat-31306_refgene -silenced plants at 120 hpi. (F) No difference in the content of Chloroplast was observed between control and wheat-36302_refgene and wheat 12266_refgene -silenced plants at 120 hpi. The error bars represent the variations among three independent replicates. The different letters represent significant differences [*P*≤0.05 according to analysis of variance (ANOVA)].

Based on the GO and KEGG pathway enrichment results, we speculated that six DEGs may play a role in the APR of XZ during *Pst* infection. Wheat37392_refgene and wheat12902_refgene were enriched in the phenylalanine metabolism pathway, whereas wheat75137_refgene and wheat31306_refgene were enriched in peroxidase activity and nitric-oxide synthase activity, and wheat36302_refgene and wheat12266_refgene were enriched in the chlorophyll biosynthetic process and the photosynthesis process. The qRT-PCR analysis showed that the six candidate genes were only up-regulated at the adult plant stage (Fig 5), and which showed that the six candidate genes were to be positive regulators of the APR at the adult plant stage. So, the candidate genes were only silenced in wheat at the adult plant stage using the Barley stripe mosaic virus (BSMV)-based virus-induced gene silencing (VIGS) system. The corresponding primers were designed and are listed in S2 Table. At the adult plant stage, all of the BSMV-inoculated plants displayed mild chlorotic mosaic symptoms at 13 dpi, but no obvious defects in further leaf growth were observed (Fig 6). We tested the silencing of the wheat *PDS* gene to confirm whether our VIGS system functioned correctly and obtained typical photo-bleaching at 15 dpi on the flag leaves of the plants inoculated with BSMV:PDS (Fig 6). To calculate the silencing efficiency of VIGS, qRT-PCR assays were performed to examine the relative transcript levels of the candidate genes in the flag leaves of plants infected with CYR32. Compared with the BSMV:γ-infected leaves, the transcript levels of candidate genes in knockdown plant were reduced from 59% to 71% at 24, 48 and 120 hpi (Fig 7). After inoculation with CYR32, hypersensitive reaction (HR) was observed in the flag leaves of the control plants (mock- and BSMV-γ-infected) (Fig 6). Phenotypic changes relative to the control were observed on the flag leaves of four candidate gene-knockdown plants (wheat37392_refgene, wheat12902_refgene, wheat75137_refgene and wheat31306_refgene), (Fig 6). On the contrary, no phenotypic changes were observed on the wheat36302_refgene- and wheat12266_refgene-knockdown plants (Fig 6). Furthermore, to determine wheather candidate genesis involved in host resistance, we have assayed histological changes in VIGS-silenced plants inoculated with *Pst* race CYR32 isolate. At 48 and 120 hpi with *Pst* race CYR32, we evaluated the necrotic area, as well as the hyphal length. The histological observations showed that the average of necrotic area per infection site decreased significantly at 120 hpi in knockdown wheat plants (wheat37392_refgene, wheat12902_refgene, wheat75137_refgene and wheat31306_refgene) compared with the control plant (Fig 8A); and the hyphal length increased significantly at 120 hpi (Fig 8B). In addition, the content of lignification and ROS decreased significantly at 120 hpi in knockdown plant (wheat12902_refgene, wheat75137_refgene and wheat31306_refgene) (Fig 8C and 8E); Wheat-37392_refgene or 12902_refgene -silenced plants showed a decrease in content of SA at 24, 48 and 120 hpi (Fig 8D); and the content of chloroplast did not changed (Fig 8F). The VIGS assays were reproduced similarly in three independent experiments.

## Discussion

### Transcripts exhibited transient expression patterns at the adult stage of wheat resistance to *Pst*

In the present study, we generated a cDNA library through the Illumina sequencing of mRNA isolated from leaves of adult plants of the wheat cultivar XZ inoculated with *Pst* CYR32. A previous study showed that when a sequencing throughput technique generates more than 2,000,000 tags, nearly all of the genes expressed in the sample can be identified in the expression data [33]. In our study, the number of clean reads from each of the eight treatments ranged from 578 to 639 million; therefore, the sequences should allow us to identify almost every gene involved in the plant response to *Pst* infection. For the transcriptome sequencing of seedling and adult plant samples at 0 hours post-mock-infection, 157,689 specific unigenes were identified. The transcription of genes in other species, such as Arabidopsis, maize, barley and cucumber, also accounted for a large proportion [34–36], which illustrates the importance of transcriptional regulation during biological activities. The assigned GO terms were summarized into three main GO categories: molecular function, cellular component and biological process. Based on the Nr annotation, the Blast2GO program was used to obtain the GO annotations. Under the biological process category, cellular process (32.05%) was the largest group, followed by metabolic process (32.01%). Under the cellular component category, cell (34.02%) and cell part (34.02%) were the largest groups, whereas in the molecular function category, binding (45.07%) was the largest group, followed by catalytic activity (40.84%). These similar aforementioned results showed that normal biological activity was dependent on the regulation of biological processes, which has also been shown through the transcriptome sequencing of microorganisms, plants, animals and humans [37, 38]. The DGE technique, which is based on the computational analysis of 21-bp tags derived from the 3’ ends of transcripts, has been used to generate transcriptome profiles for various species [18]. In the present study, the DGE profiles of wheat during *Pst* infection were based on the immediate direct application of the transcriptome data from the Sk-M-0 and Ak-M-0 samples. The sequenced tags of the six DGE libraries were then matched to 157,689 unigenes from our transcriptome reference database. Therefore, we used the gene cluster set to generate a tree that showed the similarities in the relative gene expression among the three time points. We only considered the transcript levels of unigenes that had increased or decreased by at least two-fold in each of the comparisons. The results indicated that the change in most of the unigenes was transient and that the unigenes were only altered at one time point during *Pst* infection. A few of the identified unigenes were long-lived, i.e., changes in their expression were sustained for two or three time points (Fig 3). This result was consistent with the findings of a study on the soybean response to Asian soybean rust controlled by the *Rpp2* gene [39] and of a study on the wheat response to *Pst* [40]. Indeed, it has been proposed that there is a transcriptional peak in the temporal pattern of transcript accumulation that occurs around the time of fungal penetration [40]. In the afore-mentioned study, this peak occurred at 24 hpi, which is consistent with the timing observed in our study. The 24-hpi time point reflects the haustorial penetration by *Pst* at approximately 24 hpi [12]. After this peak, the differential expression sharply declines from 72 to 96 hpi and then increases at approximately 72–168 hpi [39]. In our study, we observed a decrease in expression at 48 hpi, which is consistent with the results of the previous study [41], and then another increase at 120 hpi. This result indicates that the regulation response is differentially expressed (Fig 3). The question of why the majority of the up-regulated unigenes show a one-step-up and one-step-down pattern then arises. It is possible that the marked reprogramming of the transcriptome is connected to the three phases of *Pst* growth, which include a penetration phase, a parasitic phase and a sporulation phase [42, 43]. A GO term enrichment analysis was then applied to our data in the present study (S1 File), even though this tool has not been perfected and has limited coverage. Previous studies have suggested that upon pathogen recognition, plants activate complex signaling pathways that lead to a broad array of responses.

### The phenylpropanoid pathway plays an important role during the early stage of infection

Germlings of *Pst* penetrate the host cell and form a haustorium at 24 hpi [12]. At 24 hpi, the over-represented GO categories among the up-regulated unigenes were related to functions such as the L-phenylalanine biosynthetic process, the cell-wall macromolecule catabolic process, the SA catabolic process and the lignin catabolic process. Furthermore, the KEGG pathway analysis showed that the pathways of phenylpropanoid biosynthesis and phenylalanine metabolism were highly enriched at 24 hpi (S2 File). Previous studies have identified SA, lignins, flavonoids, phytoalexins and coumarins as secondary metabolic compounds that are produced by the phenylpropanoid pathway [44, 45]. The activation of this pathway has been shown to be involved in or related to plant defense [41, 46, 47]. In our data for the ‘biological process’ category, proteins associated with the L-phenylalanine catabolic process, the L-phenylalanine biosynthetic process and the response to phenylalanine were highly enriched at 24 and 48 hpi (S1 File). The production of these compounds relies on the conversion of phenylalanine to precursor substances by various enzymes, including phenylalanine ammonia-lyase and 4-coumarate-CoA ligase [48], all of which were observed to be enriched at 24 or 48 hpi (S1 File). Seven refgenes were enriched in the GO term L-phenylalanine biosynthetic process and were only regulated at 24 hpi according to the RNA-Seq data (S8 Fig). In the present study, to further characterize the function of phenylalanine metabolism during the wheat-*Pst* interaction, we used a knockdown approach to determine the role of wheat37392_refgene and wheat12902_refgene, which were found to be enriched in the phenylalanine metabolism pathway at 24 hpi in the wheat-*Pst* interaction at the adult plant stage. The histological observations of knockdown plants were consistent with the host response experiments, and which were also in parallel with the original hypothesis. The time point of haustorium formation in the infection sites of adult plant leaves was at 24 hpi [12]. These histological observations suggested that lignification in plants at the adult plant stage may arrest or retard fungal penetration or haustorium formation. SA is a primary plant defense hormone that is crucial for the activation of many plant defenses, including the induction of systemic acquired resistance and the HR [49, 50]. In the present study, we also found that low SA level reduced the accumulation of ROS in the knockdown plants (Fig 8), which was associated with HR. It has been suggested that the synthesis of SA occurs through two alternative pathways: the shikimate pathway (SP) and the isochorismate synthase-dependent pathway [51–54]. According to the data obtained for the ‘biological process’ category, proteins involved with SP, systemic acquired resistance and the SA-mediated signaling pathway were enriched at 24 hpi (S1 File). It was evident that the *Pst*-induced accumulation of SA by phenylalanine ammonia-lyase, which is an enzyme required for the SP-dependent pathway production of SA, was highly enriched at 24 and 48 hpi (S1 File). Additionally, the pathway of isochorismate synthase-dependent was not significantly regulated. Therefore, lignifications and SA maybe play significant roles in the APR to *Pst* infection. Taken together, these data suggest that during the early stage of the infection, the phenylpropanoid pathway plays an important role in resistance to *Pst*, which hinges on the ability to induce a broad defense response at the adult plant stage.

### The continuous accumulation of reactive oxygen species contributed to APR to *Pst*

ROS and nitric oxide (NO) are not only important as signaling mechanisms for defense but also thought to regulate programmed cell death through the establishment of a HR [55, 56]. During the development of a pathogen on adult plants, HR is not exhibited until 36 hpi in the inoculated leaves of the adult plants, and the host cells become increasingly necrotic and begin to lose their original shape at 48 hpi [12]. The analysis of the data revealed that some enrichments related to the ROS or NO production systems, including peroxidase activity and nitric-oxide synthase activity, which were highly enriched at 24 hpi, were found to be involved in the defense responses of wheat to *Pst* infection (S1 File). The ‘response to ozone’ and ‘response to hydrogen peroxide’ categories were highly enriched at 24and 48 hpi (S1 File). It was hypothesized that the first phase of ROS generation at 24 and 48 hpi coincided with the beginning of haustorium formation and subsequent HR in infected host cells. Plants attempt to maintain a dynamic balance between ROS generation and elimination through their own defense mechanisms. The ‘positive regulation of superoxide dismutase activity’ and ‘hydrogen peroxide catabolic process’ categories were highly enriched at 120 hpi (S1 File). A previous study offered similar evidence that the second burst phase of ROS at 120 hpi coincides with an increasing number of necrotic host cells and the formation of secondary hyphae surrounding the infected plant cells [12]. In this study, knockdown of wheat75137_refgene and wheat31306_refgene by VIGS decreased plant resistance to *Pst* associated with the low ROS accumulation, where oxidative stress is generated to create toxicity and kill pathogens at the infection unit. So, hyphal length increased and the necrotic area decreased in the knockdown plants (Fig 8). Therefore, we speculated that these two candidate genes most contribute to APR by modulating the ROS accumulation. Based on previous data and our study, it could be hypothesized that the burst of ROS at different phases coincides with the profiles of *Pst* development in infected plants.

### Photosynthesis was up-regulated during the later stage of infection

When responding to *Pst* infection, the wheat plant activated thousands of genes in the early stage at 24 hpi, which may induce the expression of key defense-related genes at 48 and 120 hpi through a synergistic effect. This proposed hypothesis suggests that priming or core control genes must trigger other downstream or defense-related genes through a network of pathways. By 120 hpi, there is an increasing number of necrotic host cells surrounding the attacked cells; in addition, the encased haustorial body collapses and becomes necrotic, or the haustorial body cannot expand and thus becomes necrotic [12]. The categories obtained from the GO enrichment analysis of the up-regulated unigenes at 120 hpi were related to the chlorophyll biosynthetic process, the photosystem II assembly process and photosynthetic electron transport in photosystem I, photosynthesis, light harvesting and the chloroplast relocation process (S9 Fig). In our study, we found that the unigenes involved in the glyoxylate cycle process were clearly induced at 24 hpi. In terms of primary metabolism, the metabolism of certain amino acids was also clearly altered. Several earlier studies have shown that plants appear to switch off photosynthesis locally during the early stages of the defense reaction [57]. The data obtained in this study detected no enrichments of photosynthesis at 24 and 48 hpi, which is consistent with the results of the aforementioned previous studies. This decrease was due not only to the elimination of the green (photosynthetic) leaf area as a consequence of the HR but also to an alteration in the host metabolism [58]. Photosynthesis has been reported to modulate plant defense responses induced by pathogen infection [59]. Interestingly, the photosystem II assembly process, the photosynthetic electron transport in the photosystem I process, photosynthesis, the light harvesting process and the chlorophyll biosynthetic process were enriched at 120 hpi. Surprisingly, knockdown of candidate genes (wheat36302_refgene, wheat12266_refgene) via VIGS system showed no differences compared with the control plant. There may be two main reasons why experiments failed. First, the candidate genes were only knockdown, not knockout, the silencing efficiency of the candidate genes were 61% - 76% at each time point. And second, sequence alignment with the T. *aestivum* cv. Chinese Spring genome sequence showed that there were three and four copies in the wheat genome, respectively. The starch and sucrose metabolism pathway, which is related to the carbohydrate metabolism process, was identified at 120 hpi. This proposed hypothesis is consistent with previous results: a plant’s susceptibility to certain diseases relatively depends on the sugar levels in the leaf tissues [60, 61]. Therefore, we speculate that the photosynthesis in the adult XZ plant is associated with the enhanced resistance of wheat to *Pst*.

### Thiamine metabolism was potentially linked to adult plant resistance to *Pst*

The thiamine metabolism pathway was the most enriched KEGG pathway during successive symptom phases (S2 File). Plants contain a wide range of vitamins that are essential not only for human metabolism but also for plants because of their redox chemistry and role as enzymatic cofactors in plant major metabolic pathways, including the oxidative pentose phosphate pathway, acetyl-CoA synthesis, the tricarboxylic acid cycle, the Calvin cycle, plant pigment biosynthesis, anaerobic ethanolic fermentation and the branched-chain amino acid pathway [62, 63]. In a recent study, thiamine was shown to alleviate the effects of several environmental stresses on *Zea mays* seedlings and *Arabidopsis thaliana*, presumably by protecting the plant from oxidative damage. Hence, it has been suggested that thiamine plays an indirect role as an antioxidant in plants by providing NADH and NADPH to combat oxidative stress [64, 65]. Other reported phenomena suggest that thiamine compounds may play an important role in the induction of systemic acquired resistance by activating pathogenesis-related genes in plant species against some fungal and bacterial infections [66, 67]. Thiamine is one of the products of the purine biosynthetic pathway [68], which was also found to be enriched through the KEGG pathway analysis during the infection stage (S2 File). We also speculate that the thiamine metabolism and purine biosynthesis pathways are involved in the APR to *Pst*.

## Conclusions

APR is agriculturally valuable because of its non-race specificity and durability; thus, understanding its resistance mechanism is important. In the present study, our work was carefully designed to capture the transcript response that occurs in XZ through the selection of sampling time points based on a quantitative estimation of *Pst* development. Therefore, the data obtained in this study were generated by transcriptome sequencing and digital expression profiling. It has been suggested that the change in most unigenesis is transient and that unigenes are only altered at one time point during *Pst* infection. In addition, we revealed that the development of *Pst* was markedly inhibited in adult plants at different infection stages by many biological processes, such as phenylpropanoid biosynthesis, reactive oxygen species, photosynthesis, and the thiamine metabolism pathway. Therefore, this study provides a tool for elucidating the mechanisms of APR that can be used in broader APR research.

## Supporting Information

S1 Fig Statistics of the assembly quality of the transcriptome sequencing samples. Transcriptome *de novo* assembly was performed with the short-reads assembling program Trinity, which resulted in 159,931 unigenes of Ak-M-0 and 109,606 unigenes of Sk-M-0. The two libraries included non-inoculated adult plants at 0 hours post-inoculation (hpi) (Ak-M-0) and non-inoculated seedling plants at 0 hpi (Sk-M-0). (A) The size distribution of the unigenes of Ak-M-0. (B) The size distribution of the gaps of Ak-M-0. (C) The size distribution of the unigenes of Sk-M-0. (D) The size distribution of the gaps of Sk-M-0. (E) The size distribution of all of the unigenes. (F) The size distribution of the gaps of all of the unigenes. (G) The size distribution of the ESTs obtained from the EST scan results. (H) The size distribution of the proteins predicted from the CDS sequences.
(TIF)

S2 Fig COG functional classification of all of the unigenes. A total of 157,689 unigenes showed significant homologies to genes in the COG Nr database (E-value<10^−5^) and were distributed into 25 COG categories.
(TIF)

S3 Fig GO classification of all of the unigenes. A total of 69,100 unigenes were assigned to GO term annotations using BLAST2GO and then summarized into three main GO categories and 42 sub-categories (functional groups) using WEGO.
(TIF)

S4 Fig Distribution of DEGs between the seeding and adult plant stages. All of the DEGs were obtained from this analysis: unigenes (red portion) that were up-regulated, unigenes (green portion) that were down-regulated, and unigenes (blue portion) that were not regulated at the adult plant stage. The two libraries included non-inoculated adult plants at 0 hours post-inoculation (hpi) (Ak-M-0) and non-inoculated seedling plants at 0 hpi (Sk-M-0).
(TIF)

S5 Fig Evaluation of the sequencing quality of the DGE samples. Classification of raw reads of six DGE libraries, Ak-M-24, Ak-M-48, Ak-M-120, Ak-I-24, Ak-I-48 and Ak-I-120. The six DGE libraries included non-inoculated adult plants at 24 hours post-inoculation (hpi) (Ak-M-24), 48 hpi (Ak-M-48) and 120 hpi (Ak-M-120), and inoculated adult plants at 24 hpi (Ak-I-24), 48 hpi (Ak-I-48) and 120 hpi (Ak-I-120).
(TIF)

S6 Fig Analysis of the sequencing saturation of the DGE samples. The saturation analyses of the six DGE libraries, Ak-M-24, Ak-M-48, Ak-M-120, Ak-I-24, Ak-I-48 and Ak-I-120. The six DGE libraries included non-inoculated adult plants at 24 hours post-inoculation (hpi) (Ak-M-24), 48 hpi (Ak-M-48) and 120 hpi (Ak-M-120), and inoculated adult plants at 24 hpi (Ak-I-24), 48 hpi (Ak-I-48) and 120 hpi (Ak-I-120).
(TIF)

S7 Fig Gene coverage statistics for the DGE samples. The gene coverage statistics of the DGE samples in Ak-M-24, Ak-M-48, Ak-M-120, Ak-I-24, Ak-I-48 and Ak-I-120. The six DGE libraries included non-inoculated adult plants at 24 hours post-inoculation (hpi) (Ak-M-24), 48 hpi (Ak-M-48) and 120 hpi (Ak-M-120), and inoculated adult plants at 24 hpi (Ak-I-24), 48 hpi (Ak-I-48) and 120 hpi (Ak-I-120).
(TIF)

S8 Fig The expression patterns of wheat refgenes for ‘phenylpropanoid’ were determined by RNA-Seq. The relative expression levels of eight candidate unigenes associated with the phenylpropanoid biological process at the infection stage were determined by RNA-Seq. The candidate unigenes were only expressed at 24 hours post-inoculation (hpi).
(TIF)

S9 Fig The expression patterns of wheat refgenes associated with the ‘chlorophyll biosynthetic process’ were determined by RNA-Seq. Thirty-five candidate unigenes were activated during the chlorophyll biosynthetic process. The relative expression level at the infection stage was determined by RNA-Seq. The unigenes were only expressed at 120 hours post-inoculation (hpi).
(TIF)

S1 File Enrichment of the genes up-regulated during *Puccinia striiformis* f. sp. *Tritici* infection. Fisher’s exact test with the Blast2GO software was used to explore the significantly enriched GO terms of the genes up-regulated during *Puccinia striiformis* f. sp. *Tritici* infection. The data included the ‘Biological process’, ‘Molecular function’ and ‘Cellular component’ categories.
(XLSX)

S2 File The up-regulated DEGs in the Ak-I-24 vs. Ak-M-24, Ak-I-48 vs. Ak-M-48 and Ak-I-120 vs. Ak-M-120. Comparisons were categorized according to Kyoto Encyclopedia of Genes and Genomes (KEGG) pathway analyses.
(XLSX)

S3 File qRT-PCR primer sequences for 30 DEGs. (DOCX)

S1 Table Statistics of the DGE profiling sample map to the reference genome. This table includes the number of six samples of reads that were of Total Reads, Total Base Pairs, Total Mapped Reads (sum of the highly repetitive and high quality reads), Perfect match (mapped to over 100 locations), < = 2-bp mismatch, Unique match (mapped to 1 location) and Multi-position match, respectively. The 6 DGE libraries included non-inoculated adult plants at 24 hours post-inoculation (hpi) (Ak-M-24), 48 hpi (Ak-M-48) and 120 hpi (Ak-M-120), and inoculated adult plants at 24 hpi (Ak-I-24), 48 hpi (Ak-I-48) and 120 hpi (Ak-I-120).
(DOCX)

S2 Table Virus-induced gene silencing (VIGS) system primer sequences for six candidate genes. This table includes twelve pairs of primer sequences for six genes, six pairs for BSMV-mediated gene silencing (S), and six pairs for qRT-PCR (Q).
(DOCX)
