# Supplementary figures and images for: Transcriptome Analysis Provides Insights into the Mechanisms Underlying Wheat Plant Resistance to Stripe Rust at the Adult Plant Stage
Source: PLoS One. 2016 Mar 18;11(3):e0150717. doi: 10.1371/journal.pone.0150717 (PMC4798760; doi:10.1371/journal.pone.0150717)

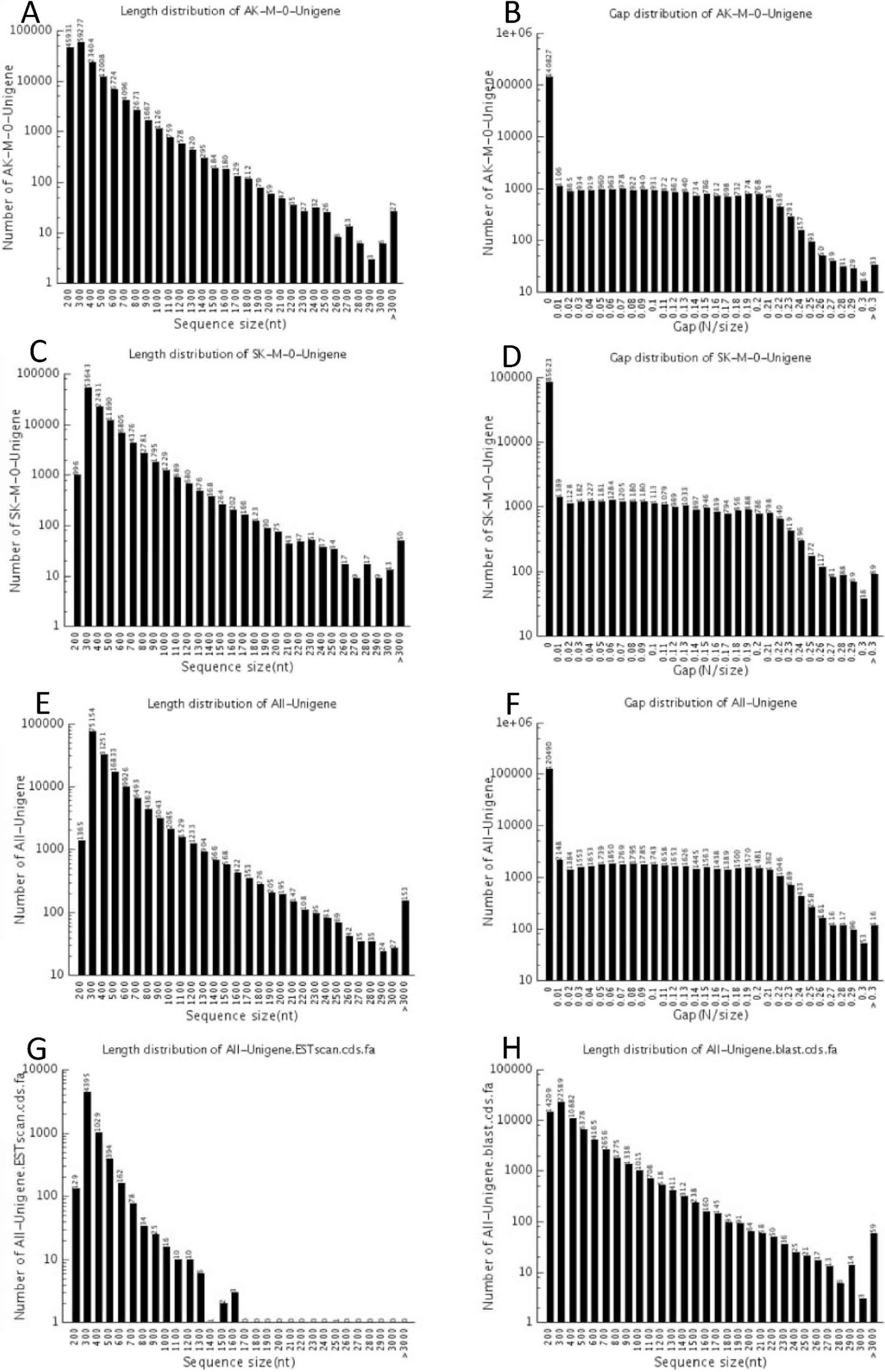

Supplement: S1 Fig — Transcriptome de novo assembly was performed with the short-reads assembling program Trinity, which resulted in 159,931 unigenes of Ak-M-0 and 109,606 unigenes of Sk-M-0. The two libraries included non-inoculated adult plants at 0 hours post-inoculation (hpi) (Ak-M-0) and non-inoculated seedling plants at 0 hpi (Sk-M-0). (A) The size distribution of the unigenes of Ak-M-0. (B) The size distribution of the gaps of Ak-M-0. (C) The size distribution of the unigenes of Sk-M-0. (D) The size distribution of the gaps of Sk-M-0. (E) The size distribution of all of the unigenes. (F) The size distribution of the gaps of all of the unigenes. (G) The size distribution of the ESTs obtained from the EST scan results. (H) The size distribution of the proteins predicted from the CDS sequences. (TIF) [file pone.0150717.s001.tif]

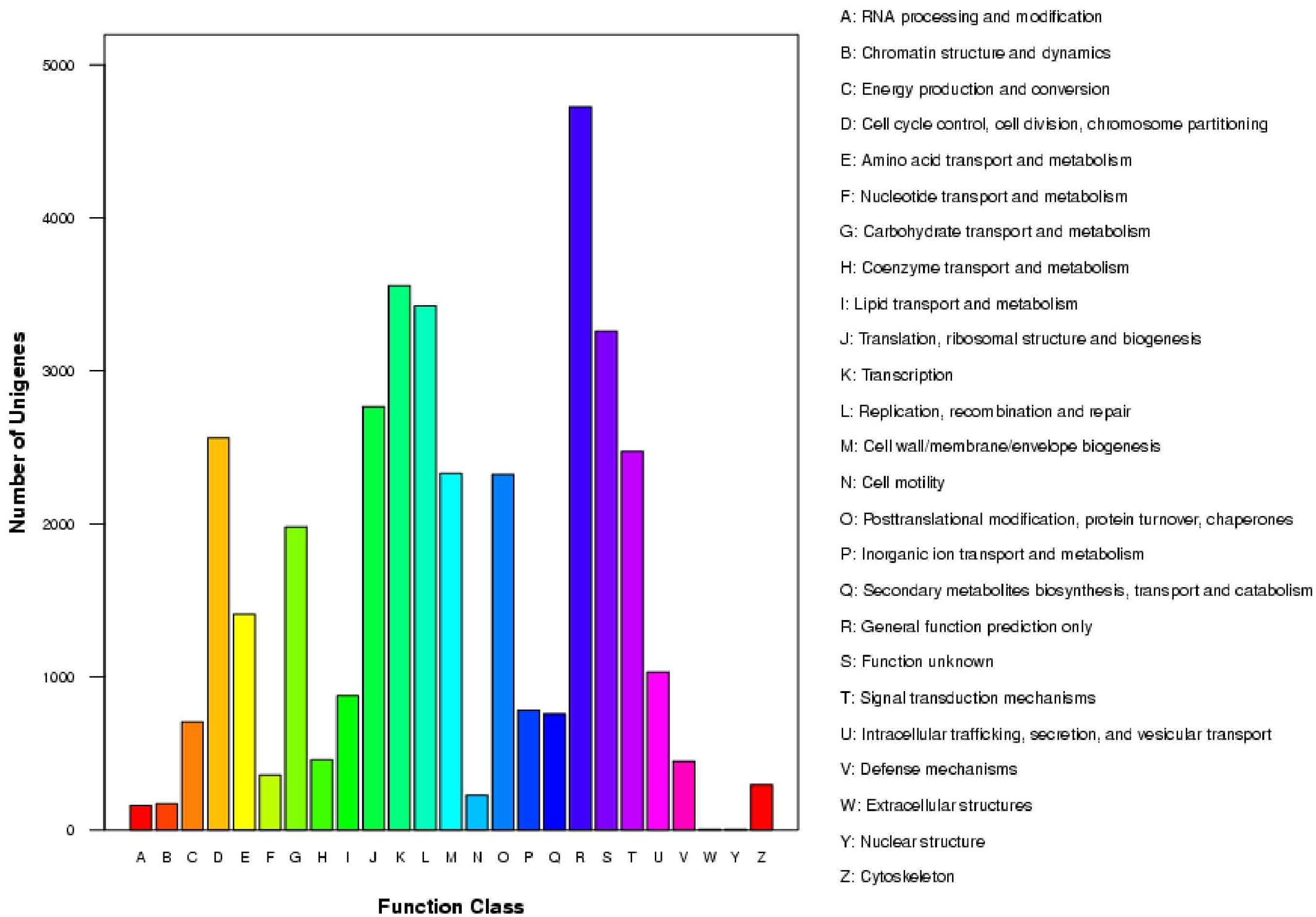

Supplement: S2 Fig — A total of 157,689 unigenes showed significant homologies to genes in the COG Nr database (E-value<10−5) and were distributed into 25 COG categories. (TIF) [file pone.0150717.s002.tif]

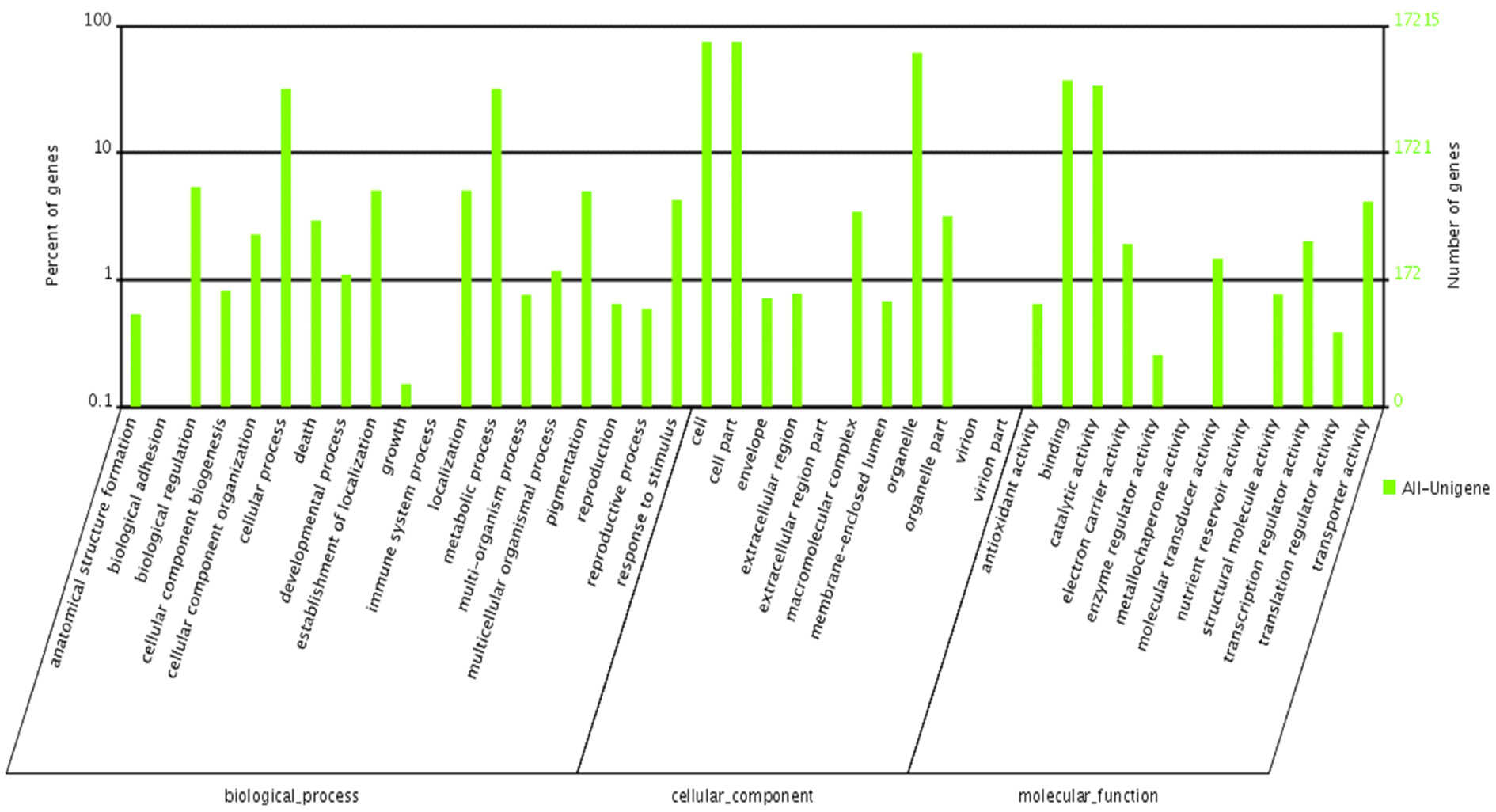

Supplement: S3 Fig — A total of 69,100 unigenes were assigned to GO term annotations using BLAST2GO and then summarized into three main GO categories and 42 sub-categories (functional groups) using WEGO. (TIF) [file pone.0150717.s003.tif]

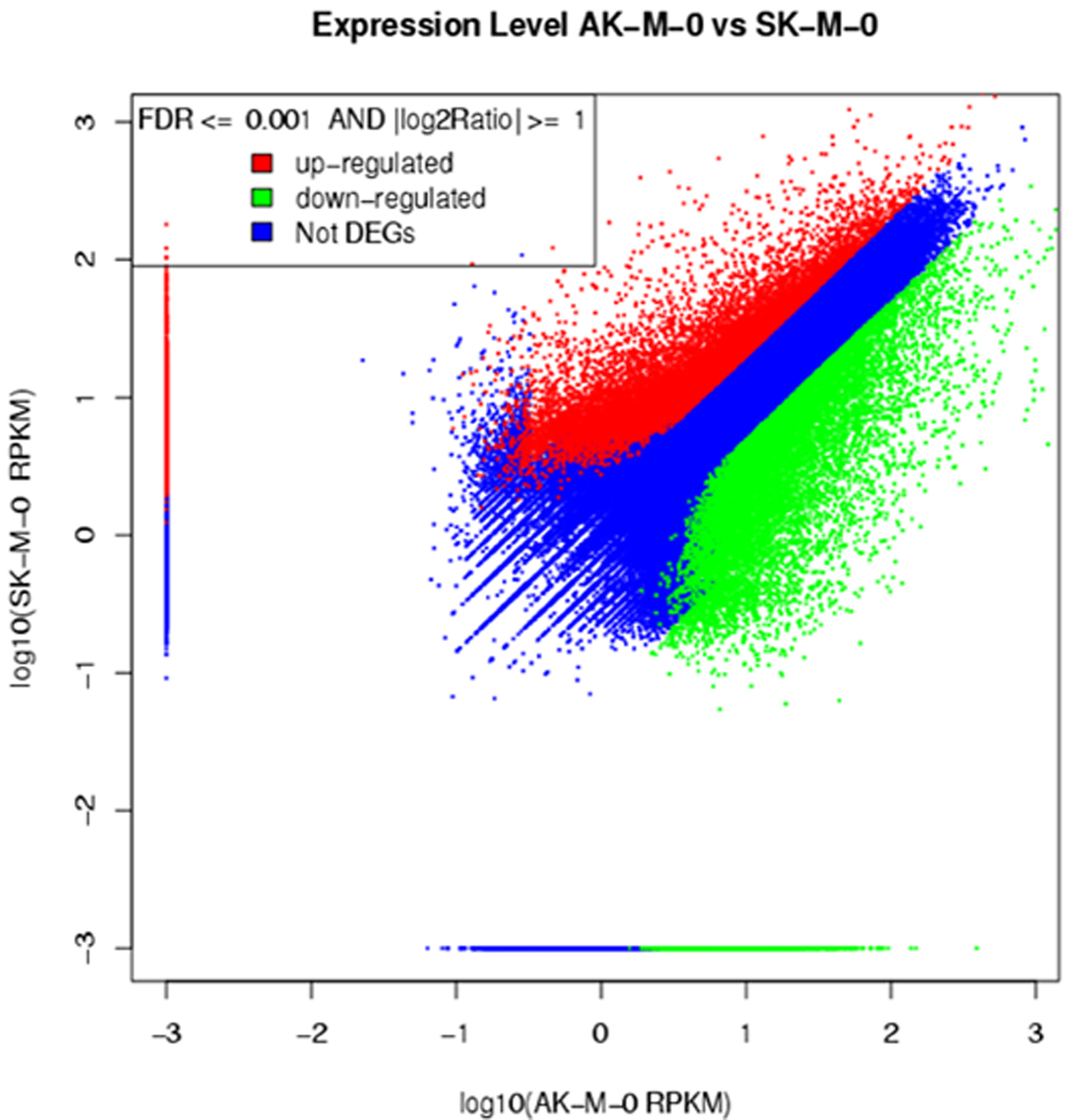

Supplement: S4 Fig — All of the DEGs were obtained from this analysis: unigenes (red portion) that were up-regulated, unigenes (green portion) that were down-regulated, and unigenes (blue portion) that were not regulated at the adult plant stage. The two libraries included non-inoculated adult plants at 0 hours post-inoculation (hpi) (Ak-M-0) and non-inoculated seedling plants at 0 hpi (Sk-M-0). (TIF) [file pone.0150717.s004.tif]

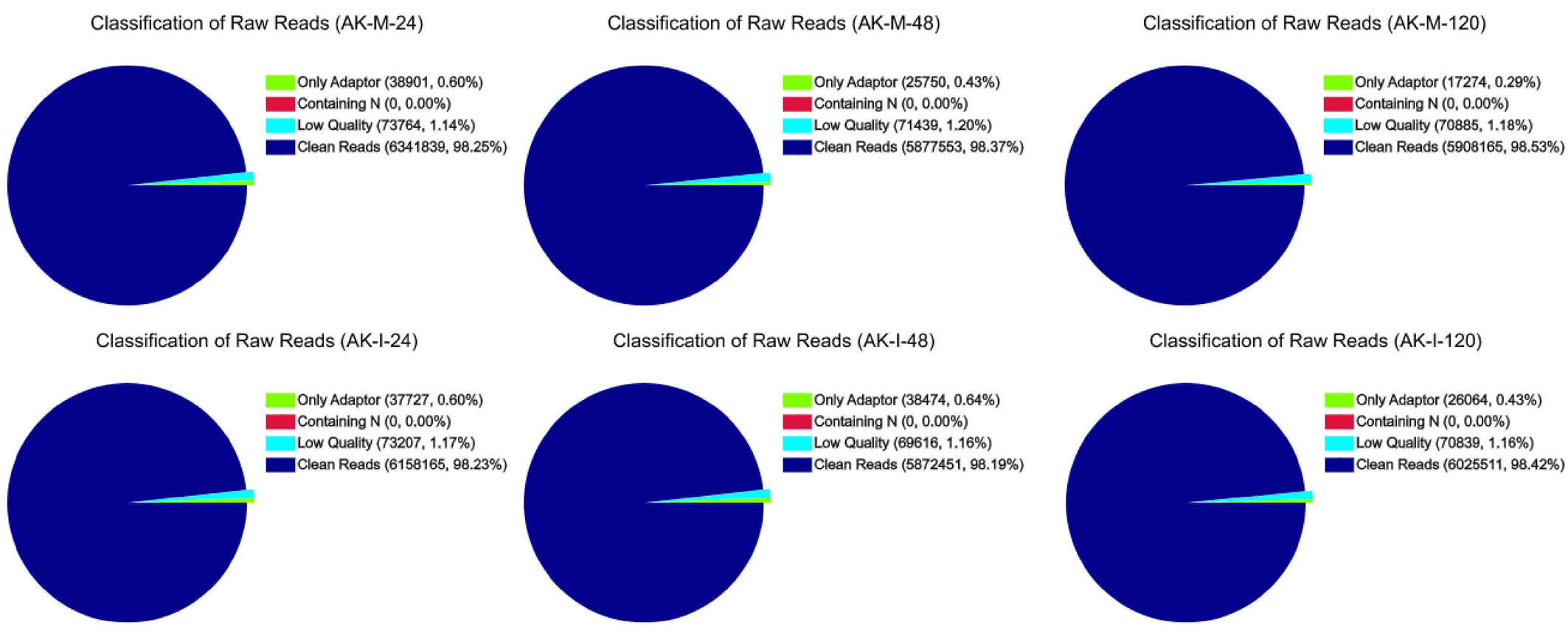

Supplement: S5 Fig — Classification of raw reads of six DGE libraries, Ak-M-24, Ak-M-48, Ak-M-120, Ak-I-24, Ak-I-48 and Ak-I-120. The six DGE libraries included non-inoculated adult plants at 24 hours post-inoculation (hpi) (Ak-M-24), 48 hpi (Ak-M-48) and 120 hpi (Ak-M-120), and inoculated adult plants at 24 hpi (Ak-I-24), 48 hpi (Ak-I-48) and 120 hpi (Ak-I-120). (TIF) [file pone.0150717.s005.tif]

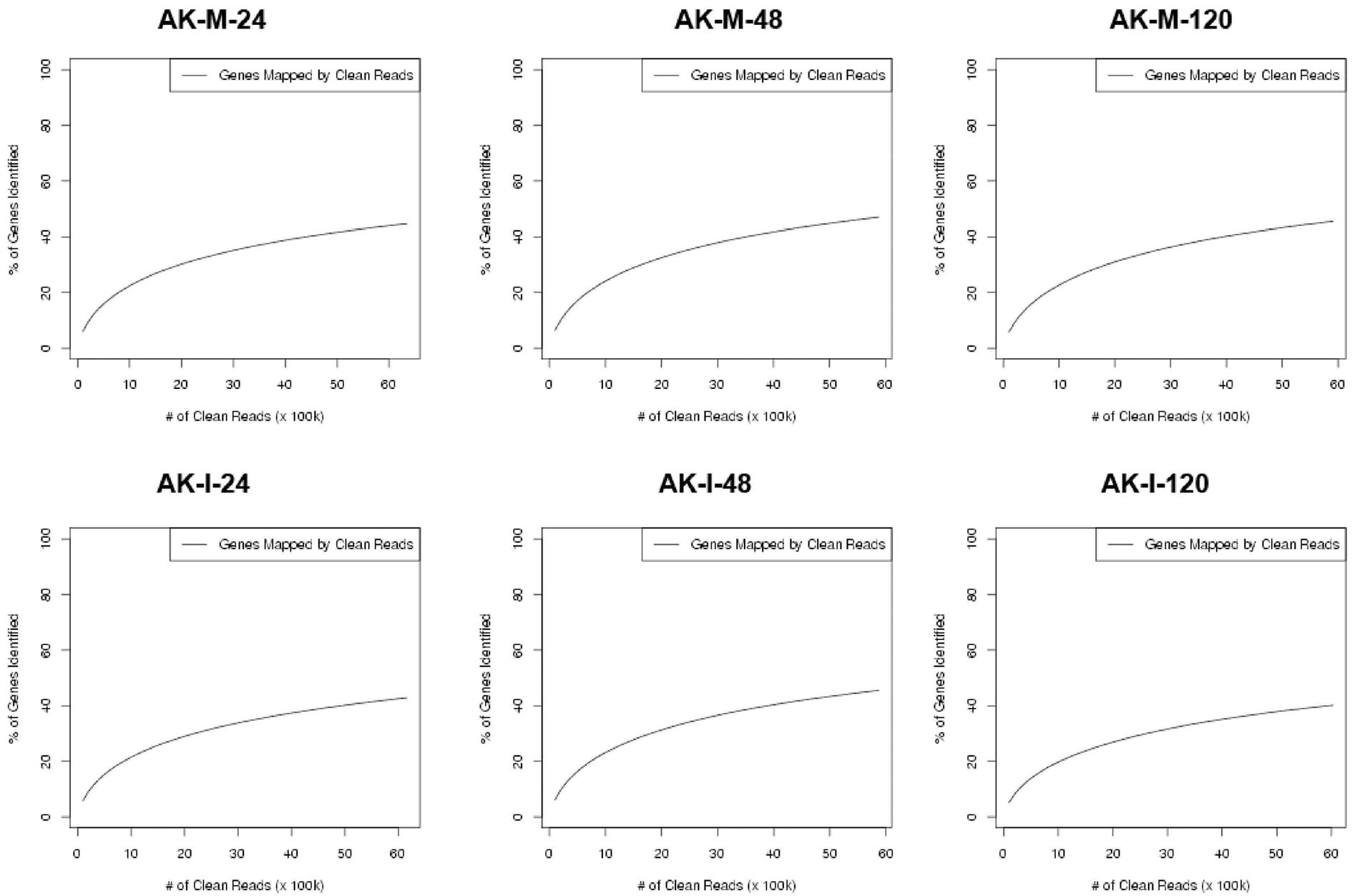

Supplement: S6 Fig — The saturation analyses of the six DGE libraries, Ak-M-24, Ak-M-48, Ak-M-120, Ak-I-24, Ak-I-48 and Ak-I-120. The six DGE libraries included non-inoculated adult plants at 24 hours post-inoculation (hpi) (Ak-M-24), 48 hpi (Ak-M-48) and 120 hpi (Ak-M-120), and inoculated adult plants at 24 hpi (Ak-I-24), 48 hpi (Ak-I-48) and 120 hpi (Ak-I-120). (TIF) [file pone.0150717.s006.tif]

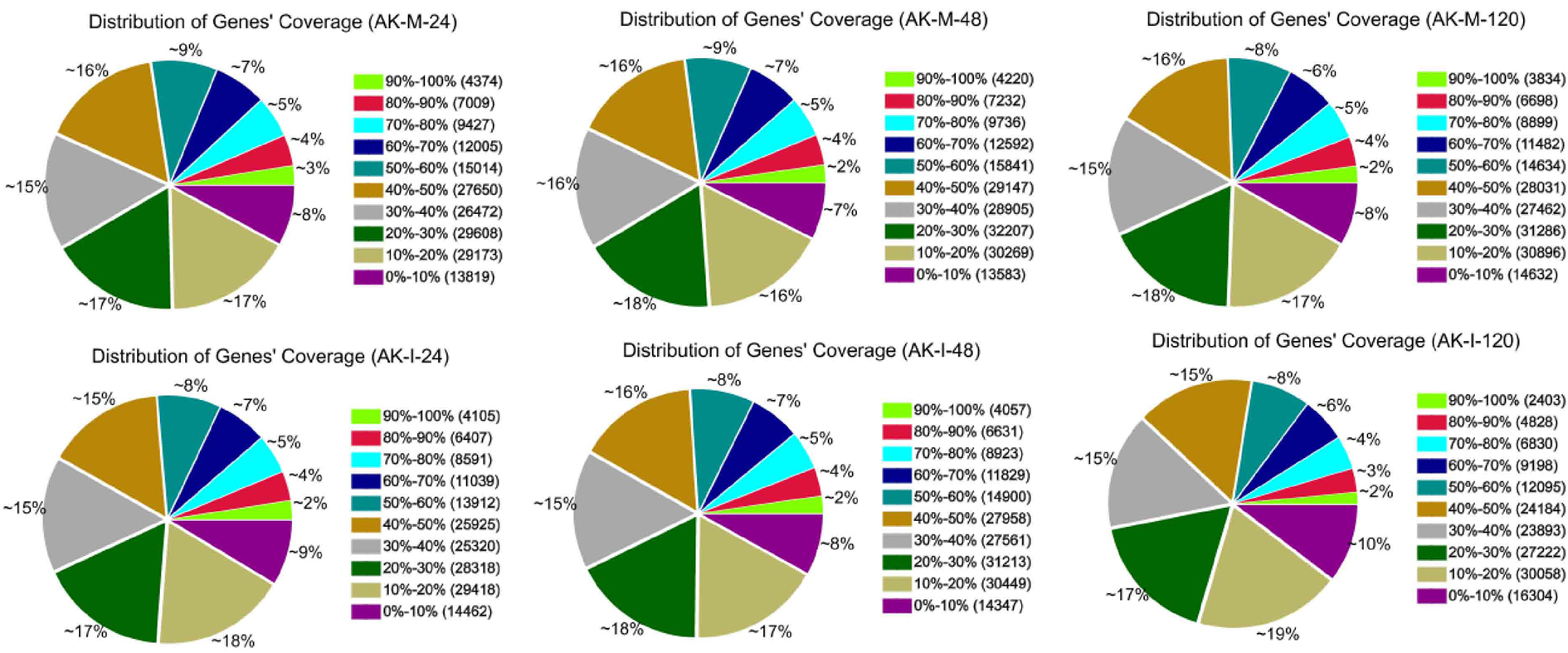

Supplement: S7 Fig — The gene coverage statistics of the DGE samples in Ak-M-24, Ak-M-48, Ak-M-120, Ak-I-24, Ak-I-48 and Ak-I-120. The six DGE libraries included non-inoculated adult plants at 24 hours post-inoculation (hpi) (Ak-M-24), 48 hpi (Ak-M-48) and 120 hpi (Ak-M-120), and inoculated adult plants at 24 hpi (Ak-I-24), 48 hpi (Ak-I-48) and 120 hpi (Ak-I-120). (TIF) [file pone.0150717.s007.tif]

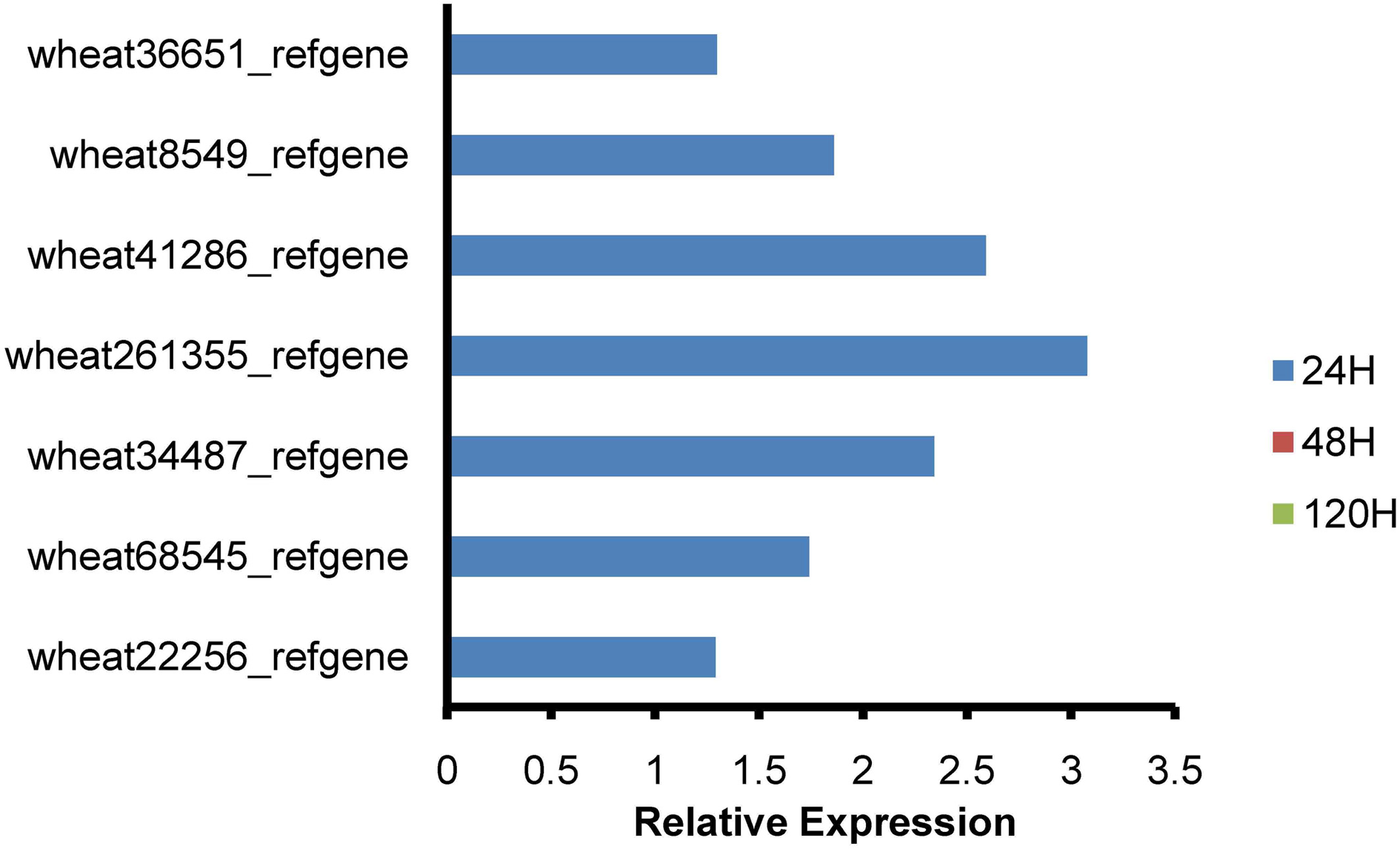

Supplement: S8 Fig — The relative expression levels of eight candidate unigenes associated with the phenylpropanoid biological process at the infection stage were determined by RNA-Seq. The candidate unigenes were only expressed at 24 hours post-inoculation (hpi). (TIF) [file pone.0150717.s008.tif]

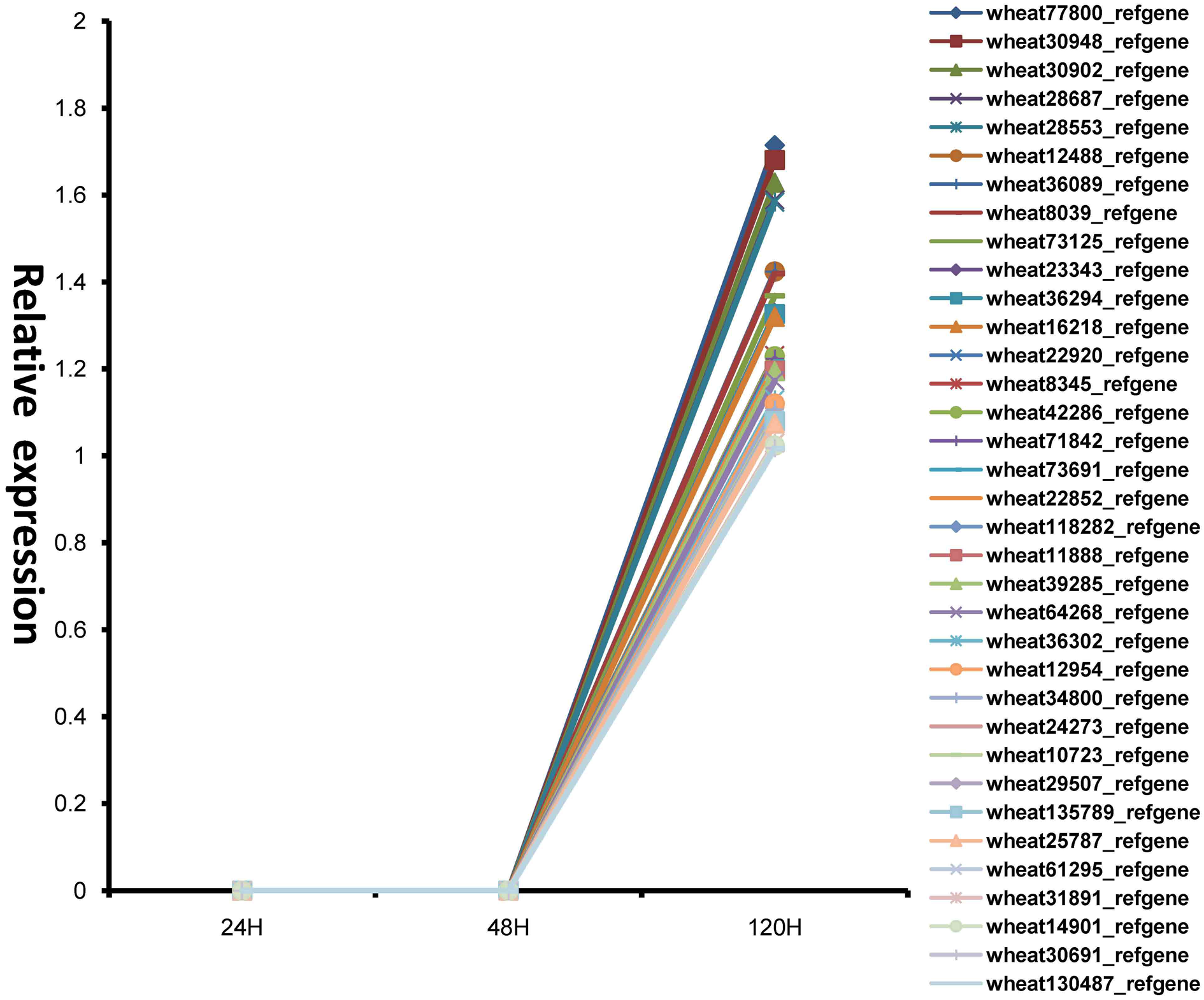

Supplement: S9 Fig — Thirty-five candidate unigenes were activated during the chlorophyll biosynthetic process. The relative expression level at the infection stage was determined by RNA-Seq. The unigenes were only expressed at 120 hours post-inoculation (hpi). (TIF) [file pone.0150717.s009.tif]
