# Supplementary material for: Transcriptome Analysis Provides Insights into the Mechanisms Underlying Wheat Plant Resistance to Stripe Rust at the Adult Plant Stage
Source: PLoS One. 2016 Mar 18;11(3):e0150717. doi: 10.1371/journal.pone.0150717 (PMC4798760; doi:10.1371/journal.pone.0150717)
Supplement: S3 File — (DOCX) [file pone.0150717.s012.docx]

**Supporting File S 3.** qRT-PCR primer sequences for 30 DEGs

| Gene Name | Primer Sequence | |
| --- | --- | --- |
|  | Forward Primer（5'-3'） | Reverse Primer（3'-5'） |
| wheat8730_refgene | TCTGCGGGATGAAGAACG | CGGACGAAATGTCGGTGT |
| wheat84218_refgene | CGTCGCACCACGAATCA | TGGTCCTTTGCCAACACG |
| wheat7284_refgene | TTCGCTGCTGTTCCTATGAG | TGATGCCCTTGGACCTGTA |
| wheat72566_refgene | GCGGCTTCAAGAACAACCA | ACGGTCCATCCATCATCACA |
| wheat33491_refgene | CACCAACTACCAGGTCATCTTCTG | ATACATCCATTTCCCGCTTACA |
| wheat31335_refgene | CAAACCTCCTGGCTGTCTT | CAGTTGACTGGACATCTAGGGT |
| wheat12328_refgene | ACGCGACCGTGTCGTTTAA | AGCCCACCGTTGATGATGTT |
| wheat121909_refgene | AGATGGCGATCTCGTCCTG | TCGGATTCCCGCTTGTTT |
| wheat10297_refgene | GTATGTGCAGCAGCAAATCAT | TGCGAGTGTAATAAGGTAGGGT |
| wheat14242_refgene | CGTGAAACACTGTCGCTCTA | ACTTACCGGGATGCCATAT |
| wheat8458_refgene | GATGGTGCCAACGATGTAG | CCATTGATGAGATCCTGCTAT |
| wheat13698_refgene | AGAAGAGTGGCGAGGATGA | CAGCGCACAAACTTAGCA |
| wheat8254_refgene | GGACGCCGAAACGCTC | TGGAACGCAACTCACAAGAC |
| wheat7959_refgene | CTGATTTCTACAGGCGATTAA | TCCAGTATTGCCACCACA |
| wheat75952_refgene | GCAACTTCATCTACGCCACCA | TCACAAACGGCTCCATCACC |
| wheat75846_refgene | GAGCCGTTCCATCTCATCT | CTTCTTCCACCTTGCTATCTT |
| wheat7310_refgene | AGATGGTGGCAATCAAGAAG | CTCAGTGGCGATGTAGACG |
| wheat70819_refgene | CCGCTTTACCAATAGTTGAGAC | CTGGAAACAGAAATACCCACAT |
| wheat66652_refgene | CCCATTGAGTCCGCAAACCCT | GTGTTATTCCCGACCGCCCTTT |
| wheat59172_refgene | TTGGCACTTTCTCGGCACG | GGGAGTCAGATGGGTTCGGTA |
| wheat57862_refgene | TGGGTTGCCGCACATT | ATGATTTAGTCCCGTTCAGGTA |
| wheat57563_refgene | AGTGGACAAGCGAGGCAATG | AGCTGTGGGACGGCGAG |
| wheat11332_refgene | ACTACGACTACGGCTCCAACA | GGCTTATTACGGCATTCCTTT |
| wheat10510_refgene | GCTGTGGGTGGACGAGAAG | CGAAGCAATCGCAGTTAGTATG |
| wheat37392_refgene | AGGCCGTAGTCACTCTGGTT | ATGCCCTCGGTATTTCACA |
| wheat12902_refgene | CGATAACGCCTACTACACCAA | GCTCTAATCACGAGTTCACCC |
| wheat75137_refgene | CCCCTGAAGCAGAACCTC | TTGAGTGAGAAGCGAATGG |
| wheat31306_refgene | TTGGGTCGTGGAGTCTGTT | CATCTCGGGCCTTCTTTT |
| wheat36302_refgene | ACCTCTACTCCGTAAAGTTCATTG | GTCGTTCCCGAGCACAAA |
| wheat12266_refgene | TCCACCACCTTGCTCATC | CTGTCGCCGAAATCCTC |
| TaEF1α | TGGTGTCATCAAGCCTGGTATGGT | ACTCATGGTGCATCTCAACGGACT |
