# Supplementary material for: Transcriptome Analysis Provides Insights into the Mechanisms Underlying Wheat Plant Resistance to Stripe Rust at the Adult Plant Stage
Source: PLoS One. 2016 Mar 18;11(3):e0150717. doi: 10.1371/journal.pone.0150717 (PMC4798760; doi:10.1371/journal.pone.0150717)
Supplement: S1 Table — This table includes the number of six samples of reads that were of Total Reads, Total Base Pairs, Total Mapped Reads (sum of the highly repetitive and high quality reads), Perfect match (mapped to over 100 locations), < = 2-bp mismatch, Unique match (mapped to 1 location) and Multi-position match, respectively. The 6 DGE libraries included non-inoculated adult plants at 24 hours post-inoculation (hpi) (Ak-M-24), 48 hpi (Ak-M-48) and 120 hpi (Ak-M-120), and inoculated adult plants at 24 hpi (Ak-I-24), 48 hpi (Ak-I-48) and 120 hpi (Ak-I-120). (DOCX) [file pone.0150717.s013.docx]

**S1 Table. Statistics of the DGE profiling sample map to the reference genome.**

**Ak-I-24** **Ak-I-48**

| **Map to Gene** | **reads number** | **percentage** |  | **Map to Gene** | **reads number** | **percentage** |
| --- | --- | --- | --- | --- | --- | --- |
| Total Reads | 5872450 | 100.00 |  | Total Reads | 6158164 | 100.00 |
| Total Base Pairs | 293622500 | 100.00 |  | Total Base Pairs | 307908200 | 100.00 |
| Total Mapped Reads | 4990125 | 84.98 |  | Total Mapped Reads | 5240716 | 85.10 |
| Perfect match | 3579847 | 60.96 |  | Perfect match | 3738090 | 60.70 |
| <=2-bp mismatch | 1410278 | 24.02 |  | <=2-bp mismatch | 1502626 | 24.40 |
| Unique match | 2525056 | 43.00 |  | Unique match | 2700645 | 43.85 |
| Multi-position match | 2465069 | 41.98 |  | Multi-position match | 2540071 | 41.25 |
| Total Unmapped Reads | 882325 | 15.02 |  | Total Unmapped Reads | 917448 | 14.90 |

**Ak-I-120**  **Ak-M-24**

| **Map to Gene** | **reads number** | **percentage** |  | **Map to Gene** | **reads number** | **percentage** |
| --- | --- | --- | --- | --- | --- | --- |
| Total Reads | 6025510 | 100.00 |  | Total Reads | 6341838 | 100.00 |
| Total Base Pairs | 301275500 | 100.00 |  | Total Base Pairs | 317091900 | 100.00 |
| Total Mapped Reads | 5327762 | 88.42 |  | Total Mapped Reads | 5463156 | 86.14 |
| Perfect match | 4034911 | 66.96 |  | Perfect match | 3934081 | 62.03 |
| <=2-bp mismatch | 1292851 | 21.46 |  | <=2-bp mismatch | 1529075 | 24.11 |
| Unique match | 2117397 | 35.14 |  | Unique match | 2717474 | 42.85 |
| Multi-position match | 3210365 | 53.28 |  | Multi-position match | 2745682 | 43.29 |
| Total Unmapped Reads | 697748 | 11.58 |  | Total Unmapped Reads | 878682 | 13.86 |
| **Ak-M-48** | | |  | **Ak-M-120** | | |
| **Map to Gene** | **reads number** | **percentage** |  | **Map to Gene** | **reads number** | **percentage** |
| Total Reads | 5877552 | 100.0 |  | Total Reads | 5908164 | 100.00 |
| Total Base Pairs | 293877600 | 100.00 |  | Total Base Pairs | 295408200 | 100.00 |
| Total Mapped Reads | 5013690 | 85.30 |  | Total Mapped Reads | 5117522 | 86.62 |
| Perfect match | 3590126 | 61.08 |  | Perfect match | 3775937 | 63.91 |
| <=2-bp mismatch | 1423564 | 24.22 |  | <=2-bp mismatch | 1341585 | 22.71 |
| Unique match | 2527249 | 43.00 |  | Unique match | 2265333 | 38.34 |
| Multi-position match | 2486441 | 42.30 |  | Multi-position match | 2852189 | 48.28 |
| Total Unmapped Reads | 863862 | 14.70 |  | Total Unmapped Reads | 790642 | 13.38 |
|  |  |  |  |  |  |  |

This table includes the number of six samples of reads that were of Total Reads, Total Base Pairs, Total Mapped Reads (sum of the highly repetitive and high quality reads), Perfect match (mapped to over 100 locations), <=2-bp mismatch, Unique match (mapped to 1 location) and Multi-position match, respectively. The 6 DGE libraries included non-inoculated adult plants at 24 hour post-inoculation (hpi) (Ak-M-24), 48 hpi (Ak-M-48) and 120 hpi (Ak-M-120), and inoculated adult plants at 24 hpi (Ak-I-24), 48 hpi (Ak-I-48) and 120 hpi (Ak-I-120).
