# Supplementary material for: Transcriptome Analysis Provides Insights into the Mechanisms Underlying Wheat Plant Resistance to Stripe Rust at the Adult Plant Stage
Source: PLoS One. 2016 Mar 18;11(3):e0150717. doi: 10.1371/journal.pone.0150717 (PMC4798760; doi:10.1371/journal.pone.0150717)
Supplement: S2 Table — This table includes twelve pairs of primer sequences for six genes, six pairs for BSMV-mediated gene silencing (S), and six pairs for qRT-PCR (Q). (DOCX) [file pone.0150717.s014.docx]

**S2 Table. Virus-induced gene silencing (VIGS) system primer sequences for six candidate genes.**

| Gene No. | Primer sequence | |  |
| --- | --- | --- | --- |
|  | Forward primer（5'-3'） | Reverse primer（3'-5'） |  |
| wheat37392_refgene | AGGCCGTAGTCACTCTGGTT | ATGCCCTCGGTATTTCACA | S  Q |
|  | CATTCAGAAGCCAAATTACTACAC | GTTCTCATCGGTTCCATTCC |  |
| wheat12902_refgene | CGATAACGCCTACTACACCAA | GCTCTAATCACGAGTTCACCC | S |
|  | TCCAGGGTGAACTCGTGAT | TGATTTGCTGCTGCTCGTA | Q |
| wheat75137_refgene | CCCCTGAAGCAGAACCTC | TTGAGTGAGAAGCGAATGG | S |
|  | ACCAAGCGCATGTCCATT | GGCGAACTGCTCGAAGAA | Q |
| wheat31306_refgene | TTGGGTCGTGGAGTCTGTT | CATCTCGGGCCTTCTTTT | S |
|  | GTCTTCAGGGCGTTCGTG | CCGCAACTGGTTTGGGTA | Q |
| wheat36302_refgene | ACCTCTACTCCGTAAAGTTCATTG | GTCGTTCCCGAGCACAAA | S |
|  | CATTAGCGGTGCGAGTTACT | CAGGCTCCCATGTCAAGAA | Q |
| wheat12266_refgene | TCCACCACCTTGCTCATC | CTGTCGCCGAAATCCTC | S |
|  | GTTGGCAAAGGCACGATAC | TGAGCAAGGTGGTGGATGT | Q |

This table includes twelve pairs of primer sequences for six genes: six pairs for BSMV-mediated gene silencing (S), and six pairs for qRT-PCR (Q).
